# Eosinophils as potential biomarkers in respiratory viral infections

**DOI:** 10.3389/fimmu.2023.1170035

**Published:** 2023-07-06

**Authors:** Iole Macchia, Valentina La Sorsa, Francesca Urbani, Sonia Moretti, Caterina Antonucci, Claudia Afferni, Giovanna Schiavoni

**Affiliations:** ^1^ Department of Oncology and Molecular Medicine, Istituto Superiore di Sanità, Rome, Italy; ^2^ Research Coordination and Support Service, Istituto Superiore di Sanità, Rome, Italy; ^3^ National HIV/AIDS Research Center, Istituto Superiore di Sanità, Rome, Italy; ^4^ National Center for Drug Research and Evaluation, Istituto Superiore di Sanità, Rome, Italy

**Keywords:** eosinophils, respiratory virus infection, COVID-19, allergic asthma, IL-33, biomarkers

## Abstract

Eosinophils are bone marrow-derived granulocytes that, under homeostatic conditions, account for as much as 1-3% of peripheral blood leukocytes. During inflammation, eosinophils can rapidly expand and infiltrate inflamed tissues, guided by cytokines and alarmins (such as IL-33), adhesion molecules and chemokines. Eosinophils play a prominent role in allergic asthma and parasitic infections. Nonetheless, they participate in the immune response against respiratory viruses such as respiratory syncytial virus and influenza. Notably, respiratory viruses are associated with asthma exacerbation. Eosinophils release several molecules endowed with antiviral activity, including cationic proteins, RNases and reactive oxygen and nitrogen species. On the other hand, eosinophils release several cytokines involved in homeostasis maintenance and Th2-related inflammation. In the context of SARS-CoV-2 infection, emerging evidence indicates that eosinophils can represent possible blood-based biomarkers for diagnosis, prognosis, and severity prediction of disease. In particular, eosinopenia seems to be an indicator of severity among patients with COVID-19, whereas an increased eosinophil count is associated with a better prognosis, including a lower incidence of complications and mortality. In the present review, we provide an overview of the role and plasticity of eosinophils focusing on various respiratory viral infections and in the context of viral and allergic disease comorbidities. We will discuss the potential utility of eosinophils as prognostic/predictive immune biomarkers in emerging respiratory viral diseases, particularly COVID-19. Finally, we will revisit some of the relevant methods and tools that have contributed to the advances in the dissection of various eosinophil subsets in different pathological settings for future biomarker definition.

## 1 Introduction

Eosinophils are a rare subset of granulocytes first observed in the peripheral blood by Wharton Jones in 1846 and subsequently named by Paul Ehrlich in 1879, based on their intracellular granules intensely stained by the acidophilic dye eosin. The role of eosinophils in the pathogenic processes was discovered only in 1922 (1), and it is currently recognized that these cells are involved in host defense, playing multiple roles in both innate and adaptive immunity (2), as well as in tissue damage and airway remodeling (3).

Eosinophils have been traditionally associated with allergic diseases, such as asthma, and parasitic infections (4–6), where these cells are able, respectively, to modulate the immune response and contribute to parasite destruction through the release of their granule-derived content (7). However, in recent years, this concept has been revised, and eosinophils are known to play a role in a wide variety of important biological processes, including regulation of homeostasis (8, 9), immune maintenance (10), glucose metabolism in adipose tissue (11), tissue regeneration (12, 13), autoimmunity (14), host defense against bacterial and viral infections (15–17), immune regulation through T helper 1 (Th1)/T helper 2 (Th2) balance modulation (18, 19) and cancer (20). Eosinophils may have, otherwise, active participation in several physiopathological mechanisms, such as exacerbation of inflammation and tissue damage, such as in some endotypes of asthma, chronic rhinosinusitis with nasal polyps, eosinophilic gastrointestinal disorders, and hypereosinophilic syndromes (14, 21, 22). Additionally, eosinophils express MHC Class II and co-stimulatory molecules and can act as antigen presenting cells (APCs) stimulating T cell responses in various compartments (23–26).

Given the sharing of target organs between respiratory allergies and respiratory viral infection diseases, in the present review we will also discuss the role of eosinophils in comorbidity conditions. In particular, we will focus on emerging or re-emerging respiratory viral infections such as severe acute respiratory syndrome coronavirus 2 (SARS-CoV-2), the cause of the COVID-19 pandemic, and influenza viruses that continue to pose significant global public health threats, highlighting the relevance of using eosinophil counts as immune/clinical biomarkers.

## 2 General biology of eosinophils: development, effector mechanisms and heterogeneity

Eosinophils originate in the bone marrow through a series of progenitors and mature to the final stage driven by transcription factors (i.e., GATA-1, C/EBPα, PU.1, and XBP1) and by the action of cytokines (i.e., IL-3, IL-5 and GM-CSF), which enable their maturation and migration in the bloodstream. The migration is also sustained by IL-5 secretion produced by type 2 innate lymphoid cells (ILC2s), where both ILC2s and eosinophils are activated by epithelial-derived alarmins, such as IL-33 (27). Once released into the peripheral blood, eosinophils have a limited life span (∼18 hours) and only a low number of circulating eosinophils can be detected (< 450–500 eosinophils/µL) (28). Under homeostatic conditions, eosinophils rapidly migrate into the adipose tissue, thymus, lungs, uterus, mammary glands, and particularly into the gastrointestinal tract, where they are predominantly involved in the specific physiology of each tissue or organ (29–32). In response to inflammatory stimuli to the chemokines CCL11 (eotaxin-1), CCL24 (eotaxin-2), and CCL5 (RANTES), eosinophils migrate to inflamed tissues or to sites of infection where their survival is prolonged (33).

Eosinophil granules contain cationic proteins, such as major basic protein (MBP), eosinophil cationic protein (ECP), eosinophil peroxidase (EPO), and eosinophil-derived neurotoxin (EDN) endowed with cytotoxic antiparasitic and antibacterial functions. In addition, eosinophils may release a variety of mediators, including cytokines, chemokines, enzymes and lipid mediators either soluble or stored in vesicles, including exosomes, that are released in the extracellular space in response to a variety of stimuli (7). Even though eosinophils are frequently associated with Th2 responses, their granules contain preformed IL-2, IL-12, and IFN-γ (34, 35) which are typical Th1 cytokines. Thus, besides exerting a well-established role in fighting parasites, eosinophils are also involved in the host defense against fungi, bacteria and viruses (36).

Emerging evidences indicate that the functional plasticity of eosinophils may reflect the existence of different eosinophil subsets, supporting the hypothesis that the microenvironment can modulate the activity of eosinophils (37). In fact, the local microenvironment is capable of inducing changes in eosinophil phenotype depending on specific functions of the tissue. Two main subtypes of eosinophils have been described based on phenotype, morphology, functions, response to IL-5 and organ location: resident eosinophils with homeostatic function (rEos) and inflammatory eosinophils (iEos). In mice, the normal lung contains rEos described as IL-5-independent parenchymal Siglec-F^int^CD62L^+^CD101^lo^ cells with a ring-shaped nucleus. During house dust mite (HDM)–induced airway allergy, the lung contained both rEos and recruited inflammatory eosinophils (iEos), defined as IL-5-dependent peribronchial Siglec-F^hi^CD62L^–^CD101^hi^ cells with a segmented nucleus (38). The first subset usually expresses CCR3, Siglec-F, and CD125 (39) while iEos exhibit high levels of CD11b, F4/80, CD69, and CD44 (40). Therefore, the heterogeneity of eosinophils in phenotype and function depends on maturation, location and microenvironment. Similarly, rEos found in the human lung of non-asthmatic subjects (Siglec-8^+^CD62L^+^IL-3R^lo^ cells) were phenotypically distinct from the iEos isolated from the sputum of eosinophilic asthmatic patients (Siglec-8^+^CD62L^lo^IL-3R^hi^ cells) (38). Human circulating eosinophil characterization is described more in detail below.

## 3 Eosinophils in respiratory allergies

Respiratory allergies include several syndromes, in which characteristic acute symptoms represented by asthma and/or rhinitis are rapidly induced by inhalation of apparently innocuous airborne substances called allergens. Main pathophysiological mechanism of respiratory allergy, is represented by upper and/or lower airways inflammation due to a dysregulated immune response toward the allergens, based on Th2 lymphocytes (41, 42). Asthma phenotype is characterized by bronchial hyperactivity, airflow obstruction, and airway remodeling, although not all of these clinical hallmarks of allergic asthma are present with similar frequency and intensity in all patients. In fact, in the last years, the classification of asthma phenotypes has evolved into several asthma endotypes, defined by underlying pathophysiological mechanisms, which might lead to direct differences in responsiveness to common therapies, such as inhaled corticosteroids or specific biologicals (43). For example, an anti-eosinophilic medication (anti-IL-5 monoclonal antibody) did not meet its primary or secondary endpoints in all-comers trials, but clinical efficacy became apparent when targeted to patients with increased blood and sputum eosinophil counts (43). As such, the type 2-high allergic asthma endotype is orchestrated by Th2-associated cytokines such as IL-4, IL-5 and IL-13, into different sub-endotypes ranging from mild to very severe form of the diseases (44).

One of most relevant markers both for allergic asthma and allergic rhinitis is blood eosinophilia. Importantly, both blood and airways eosinophilia is considered a relevant marker for endotype-driven treatment of allergic asthma according to a recently developed patient-tailored therapy approach (45). All secreted Th2 cytokines are involved in eosinophil recruitment, migration or survival and are responsible for the increased eosinophil numbers in bronchoalveolar lavage fluid (46). Moreover, lipids such as leukotriene E4, Platelet activating factor (PAF) and prostaglandin D2, or specific chemokines called eotaxins (CCL11, CCL24 and CCL26) are able to directly recruit eosinophils to the airways. Of note, not only adaptive mechanisms induce eosinophil-chemoattractive substances, but also innate immune mechanisms, mainly mediated by epithelial-derived cytokines (IL-33, IL-25, TSLP), are involved in airways eosinophil recruitment (47). When activated, eosinophils can promote their own survival in the tissue by autocrine secretion of IL-5, which inhibits eosinophil apoptosis (48), and of GM-CSF and IL-3 upon adherence to fibronectin (49), allowing for long term persistence of eosinophils at inflammatory sites.

Eosinophils play a prominent role in the development of clinical allergic asthma hallmarks. First, they damage airways epithelium by releasing reactive oxygen species and hazardous molecules such as MBP, EPO and ECP (50) which, in turn, promote bronchial hyperesponsiveness (51). Then, eosinophils promote epithelial to mesenchymal transition of bronchial epithelial cells by secreting TGF-β (52), smooth muscle cells proliferation and collagen deposition (53) also by delivery of exosomes to structural lung cells (54).

Airways remodeling is a process that can occur physiologically as a tissue reparative mechanism, where eosinophils have a critical homeostatic role (55). However, depending on chronic inflammation and/or genetic alteration of respiratory epithelium, the remodeling process may induce fibrosis, a process that represents the most important pathological component of severe asthma (56).

## 4 Role of eosinophils in respiratory viral diseases

**Figure 1:**
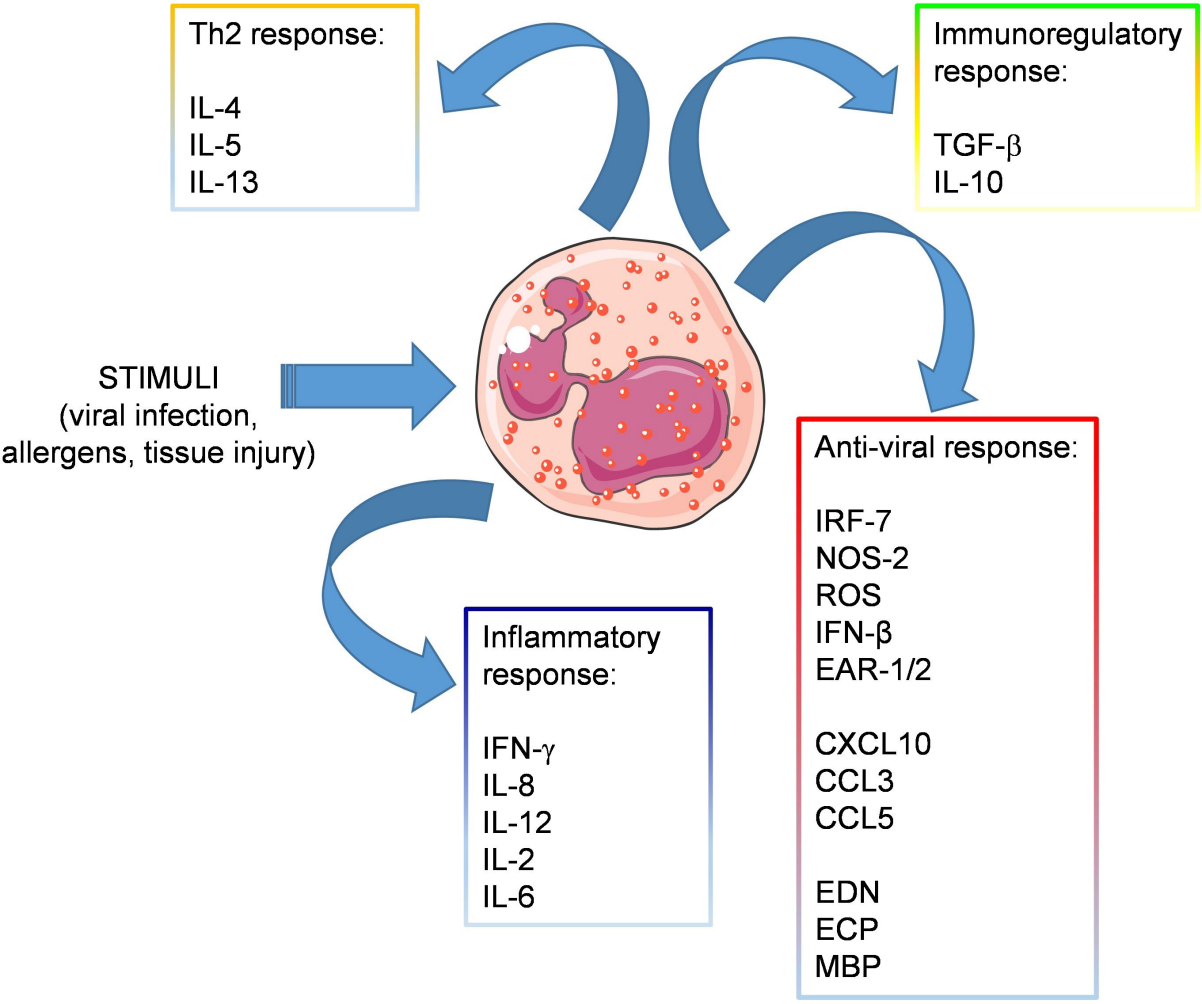
Release of soluble mediators by eosinophils. Eosinophils respond to stimuli released in the context of viral or allergic sensitization, by producing various soluble mediators involved in: inflammation (IFN-γ, IL-8, IL-12, IL-2, IL-6), Th2 response (IL-4, IL-5, IL-13), immune regulation (TGF-β, IL-10) and antiviral response, including antiviral factors (IRF-7, NOS-2, IFN-β, and the ribonucleases EAR-1 and EAR-2), chemokines attracting T cell effectors (CXCL10., CCL3, CCL5) and granule-derived cationic proteins (EDN, ECP, MBP).

Eosinophils are present in the airways and under homeostatic conditions contribute to the maintenance of lung immune homeostasis. During viral infections, eosinophils may participate directly or indirectly to antiviral immune responses through production of various soluble mediators (**Figure 1**) and can exert beneficial effects against respiratory viruses as long as they do not induce a detrimental inflammatory response in the airways, such as during allergic asthma.

Eosinophils contain molecules in their granules endowed with potential antiviral activity, including ribonucleases (e.g. eosinophil-derived neurotoxins and eosinophil cationic proteins), nitric oxide (NO) and several cytokines, which promote antigen presentation and enhance CD8^+^ T cell response (21, 57). Eosinophils express toll-like receptors that are engaged during viral recognition, such as TLR-3 (58, 59) TLR-7, and TLR-9 (60). In particular TLR-7, which recognizes single-stranded RNA (ssRNA) (61), is expressed at higher levels than in neutrophils, and signaling through the receptor increases the expression of adhesion molecules in eosinophils such as L-selectin and CD11b, induces the generation of superoxide anions, and promotes survival after activation by IFN-γ (59, 61, 62). Moreover, eosinophils express receptors for viral pathogen sensing. Thus, expression of Retinoic acid-inducible gene I (RIG-I) by eosinophils enables the recognition of RNA sequences marked with 5′ triphosphorylated ends that could trigger the immune response (63). Moreover, eosinophils can recognize products of cell necrosis, such as High Mobility Group Box 1 (HMGB1), through its specific receptor (i.e., RAGE), resulting in eosinophil degranulation, oxidative burst and amplification of the inflammatory response (27, 64, 65).

In response to viruses, eosinophils can secrete IFN regulatory factor 7 (IRF7), NOS-2, IFN-β, ribonucleases (i.e., EAR-1 and EAR-2), and several interleukins and chemokines (i.e., IL-6, IP10, CCL2, and CCL3), all of which have variable effects on viral clearance (59, 66, 67). ECP, for instance, not only exhibits antibacterial and antiparasitic activities, but it is also a member of the ribonuclease A family along with EDN (68). It has been demonstrated that both proteins have an RNase activity (69, 70), so they could play a role against ssRNA viruses. Eosinophils are also capable of producing oxidizing species through EPO and to produce NO by inducible NO synthase, a molecule that inhibits viral replication by multiple mechanisms and which is effective against several viruses (71).

Similar to neutrophils, eosinophils are able to produce extracellular traps (ETs) (72). While neutrophil ETs have been reported to exert antiviral effects (73), it is currently unknown whether this is the case also for eosinophil ETs. Finally, in response to different antigens including viral antigens, eosinophils can act as APC, expressing CD80, CD86, CD28, and CD40 (74–76) and are able to migrate to lymph nodes where they can stimulate T cell immunity (74, 77).

Eosinophils are known to function as a versatile coordinator that actively regulates or interacts with various immune cells including T lymphocytes and dendritic cells (2). As an example, in the context of viral infections, eosinophils pulsed with influenza peptides are able to activate antigen-specific T CD8^+^ cells, inducing the release of IFN-γ and TNF-α. Human rhinovirus binds to eosinophil ICAM-1 and induces antigen-specific T CD4^+^ proliferation and IFN-γ release; otherwise, human influenza virus and parainfluenza virus replication in eosinophils is abortive (78). In settings other than viral infections, eosinophils can play a role in in the T-cell selection (79). Furthermore, eosinophils-mediated antigen priming of B cells triggers antigen-specific IgM production and eosinophils can suppress Th17 differentiation *via* IL-1R release (in the intestine). Eosinophil-derived CXCL5 can directly activate neutrophils, while eosinophils IL-13 and IL-14 secretion can influence macrophages in adipose tissues by inhibiting inflammation. CpG DNA-stimulated eosinophils can release EDN and induce DC maturation. Locally, intestinal eosinophils release EPO to activate DCs and trigger their migration to draining lymph nodes. Also, EPO is positively involved in the regulation of macrophage phagocytosis (80).

### 4.1 Respiratory Syncytial Virus

Respiratory Syncytial Virus (RSV) is one of the most important pathogens that causes airway infections during childhood (81). Approximately 50% of pneumonia cases in adult and elderly patients (82) and up to 90% of bronchiolitis cases during childhood are caused by this virus (83). RSV is an enveloped virus that contains a non-segmented, negative-sense ssRNA. The role of eosinophils in the antiviral response to this virus was initially controversial but the fact that ssRNA is able to induce EPO release and degranulation suggested the possible participation of these granulocytes to RSV response (59). *In vitro* studies demonstrated that exposure of eosinophils to RSV infected epithelial cells or TLR7 ligands up-regulates the activation marker CD11b on eosinophils and triggers ECP release. In turn, ECP and EDN exerted an antiviral effect due to their RNAse activity (59, 62). In addition, production of NO has been postulated as one of the most important elements in the antiviral mechanisms by which eosinophils lower the viral titer, with these effects probably depending on TLR-7 engagement and MyD88 adaptor protein–dependent signaling (84, 85).

Dyer and co-Workers reported that human and mouse bone marrow-derived eosinophils can be infected by RSV and the closely related mouse pneumonia virus (PVM), respectively, inducing the release of IL-6 and, in mice, also the release of CXCL10, CCL2, and CCL3 related to monocyte chemoattraction and macrophage activation under inflammatory conditions. In addition, eosinophils from MyD88 deficient mice displayed reduced release of IL-6 and an accelerated PVM replication indicating that the antiviral effect could depend on MyD88 signaling (67).

Studies on transgenic (Tg) models infected with RSV found an accelerated viral clearance in hypereosinophilic (IL-5 Tg) mice while eosinophil-deficient mice showed a reduced viral clearance. Interestingly adoptive transfer of MyD88-sufficient, but not MyD88-deficient eosinophils into RSV-infected wild-type (WT) mice demonstrated that eosinophils accelerated viral clearance *via* MyD88-dependent pathways (59). Moreover, hypereosinophilic eotaxin-2/IL-5 double Tg mice infected with PVM presented a reduction in the virus recovered from lungs compared with the control strain, and were protected from a lethal inoculum of PVM (86).

Contradictory results have been reported related to the role of eosinophils in murine models of vaccine enhanced disease (VED) or immunopotentiation associated with RSV. Different vaccine formulations against RSV showed the presence of eosinophils to be an important element of inflammatory status and linked to poorer disease progression, findings which are typical in VED associated with RSV. Pennings and co-Workers performed blood mRNA transcriptome analysis during VED in RSV vaccinated mice and observed an increase in expression of EAR-1/2/3/6, which are associated with eosinophils (87). In contrast, some authors reported that eosinophils could play a dual or even protective role in RSV infection by avoiding the VED. For instance, Su and co-Workers (88) using eotaxin and IL-5 double-knockout (EotIL5-/-) mice, devoid of eosinophils in blood and lungs, demonstrated that EotIL-5-/- mice immunized or not with a RSV vaccine (virus-like nanoparticles carrying RSV fusion proteins: FIRSV), presented higher viral titers after RSV challenge compared with controls. Adoptive transfer of eosinophils from IL-5 Tg to FIRSV-immunized and RSV-infected EotIL-5-/- mice led to a reduced viral titer and an increment in IFN-β production, compared to animals not receiving eosinophils. In contrast, two studies suggested that eosinophils are not necessarily a critical immune component associated with immunopotentiation linked to RSV vaccine administration that seems primarily to be mediated by CD4+ T cells instead (89, 90).

### 4.2 Human influenza virus

Influenza viruses are enveloped, negative-sense single-stranded segmented RNA viruses, that are common causes of human respiratory infections, whose severity ranges from mild to lethal (91). Eosinophils are not regarded as the main effector cells in the antiviral immune response; despite this, epidemiological data collected during the 2009 H1N1 pandemic suggested that patients with asthma, probably owing to pulmonary eosinophilia, had less severe outcomes associated with viral infection compared with non-asthmatics individuals (92–95).

In a combined murine model of acute allergy and influenza infection, Samarasinghe and co-Workers showed that mice displayed higher numbers of eosinophils in the airways and an accelerated virus clearance compared with infected mice with chronic asthma. Moreover, mice with acute asthma also presented higher numbers of CD8^+^ T cells and minor epithelial damage suggesting that eosinophils confer protection from Influenza A virus (IAV)-induced airway damage (96, 97). The same group demonstrated that adoptive transfer of eosinophils from asthma allergic mice into the airways of IAV-infected mice reduced viral burden and increased CD8^+^ T cell numbers in the airways. *In vitro*, mouse eosinophils were susceptible to IAV infection, inducing piecemeal degranulation (producing several cytokines and soluble mediators, such as NO) and an overexpression of MHC-I and CD86. Furthermore, virus-pulsed eosinophils were able to induce CD8^+^ T cell responses (77). These findings suggest that eosinophils have the capacity to stimulate virus-specific CD8^+^ T cell responses by serving as APCs and as a source of cytokines in the lung microenvironment thus protecting from IAV infection. Notably, IAV infection could be abortive in eosinophils and this constitutes a passive mechanism exerted by these cells to limit viral expansion (36, 77). The above mentioned studies support the evidence that eosinophils could be important mediators in immunity to influenza virus.

### 4.3 Human parainfluenza virus

Human parainfluenza virus (PIV) is an enveloped, negative-sense single-stranded non-segmented RNA virus that gives rise to lower respiratory infections in infants, elderly people and immunocompromised patients, and have been detected in children with acute asthmatic exacerbations (98, 99). In a similar fashion, as for the other respiratory viruses, eosinophils seem to exert their antiviral action through the TLR-MyD88 pathway involvement (59). Adamko and co-Workers reported that eosinophils could play an antiviral role during PIV infection by reducing viral content in the lungs, which was reverted by using anti-IL-5 antibodies, suggesting that the observed effect originated by the recruitment of eosinophils to the lungs (100).

Other studies used an *in vivo* model and *in vitro* human eosinophils to evaluate the antiviral role of eosinophils against PIV. PIV infected NJ.1726 IL-5 transgenic mice, which are characterized by the accumulation of bronchial eosinophils, presented a viral RNA reduction compared with controls strains (101). Drake and co-Workers demonstrated that human eosinophils had antiviral activity versus PIV *in vitro*, and this activity increased when eosinophils were pre-incubated with IFN-γ. This antiviral effect was mostly mediated by NO generation through TLR-7, while eosinophilic RNases did not seem to play a role (102). The same group stated that human peripheral blood eosinophils from healthy volunteers were susceptible to PIV infection, but the viral progeny was not infectious, suggesting that abortive infection, as with influenza virus, constitutes one of the mechanisms by which these leukocytes restrain viral expansion.

### 4.4 Human rhinovirus

Human rhinovirus (HRV) is a positive-sense, ssRNA virus frequently identified in upper respiratory tract infections and it is associated to acute asthmatic exacerbations, mainly in childhood, severe bronchiolitis in infants as well as in fatal pneumonia in elderly and immunocompromised adults (103). The antiviral activity generated by eosinophils in this context seems to be mediated by their binding to HRV-16 through ICAM-1 and their behavior as APCs inducing CD4^+^ T-cell proliferation and IFN-γ production. The latter might then increase the expression of TLR-7 and TLR-8 on eosinophils suggesting a cooperation between eosinophils and T cells (104).

Of note, in asthmatic patients, eosinophils exhibit a reduced capacity to bind to viruses, and HRV induces a perturbation in asthma control strongly correlated with a diminished CD69 expression on the surface of these granulocytes (105). However, depletion of eosinophils (although incomplete) as a consequence of treatment with mepolizumab (a humanized monoclonal antibody anti-IL-5) followed by challenge with HRV-16, resulted in an enhanced viral titer, thus proving the relevance of eosinophils to counteract the viral respiratory infection (106). These contrasting results suggest that eosinophils could be regarded as a double-edged sword, leading to an excessive immune response in the attempt to eliminate the virus that causes damage to the host (107). More in-depth knowledge of the role and mechanisms of eosinophils in different contexts is still required.

## 5 Eosinophils and COVID-19: recent insights

### 5.1 Eosinophils as potential biomarkers associated with COVID-19 severity

Since the discovery of the Coronavirus Disease 2019 (COVID-19), caused by the SARS-CoV-2, many key questions were raised on the potential relationship between eosinophil count and the clinical course and severity of disease (16). SARS-CoV-2 infected patients present diverse clinical profiles, ranging from asymptomatic to severe respiratory failure and death. Early detection of high-risk patients is therefore fundamental to tailor therapeutic interventions that anticipate disease progression and prevent poor outcomes. For this reason, the identification of biomarkers, such as cellular and molecular mediators of immune response, could contribute to the prognosis and management of COVID-19 patients (36). The immune patterns of COVID-19 include lymphopenia, lymphocyte activation and dysfunction, increased production of cytokines, especially of IL-1β, IL-6, and IL-10, increased IgG antibodies as well as elevated levels of C-reactive protein (CRP). Additionally, eosinopenia correlates with biomarkers of coagulation disorder and those of tissue damage in kidney, liver, and other tissues (108). Namely, neutrophil levels are significantly higher in severe patients, while the percentage of eosinophils, basophils, and monocytes are reduced (109).

The current literature shows that the peripheral blood eosinophil count (EC) could be regarded as a possible predictive and prognostic biomarker for clinical outcome (110–112). The EC in the body is normally tightly regulated and accounts for only a small minority of peripheral blood leukocytes (1-3%). Normal EC ranges from 200 x 10^3^/μL to 520 x 10^3^/μL. Peripheral blood eosinophilia (≥500 x 10^3^/μL) may be caused by numerous conditions, including allergic, infectious, inflammatory, and neoplastic disorders whereas eosinopenia is defined as a reduction of circulating eosinophils < 10 eosinophils/μL and may be somewhat more difficult to recognize. Of note, eosinopenia is not pathognomonic for any disorder or clinical state. Many clinical conditions have been associated with eosinopenia, including a wide variety of virus infections, such as SARS-CoV-2 (113).

Variable EC have been reported during SARS-CoV-2 infection. However, whether these changes are related to the primary disease process or due to immunomodulation by the used treatment is not clear (114). Notably, a profound and persistent eosinopenia has been related to SARS-CoV-2 infection and associated with clinical worsening and increased risk of mortality (18, 111). Blood eosinopenia has been identified as one of the earliest indicators of severity among patients with COVID-19 (36) and severe eosinopenia in hospitalized COVID-19 adult patients may reflect the magnitude of immune hyperactivation during severe-to-critical COVID-19 (115). Persistent eosinopenia and lymphopenia were associated with the cytokine storm, which appeared in patients with pulmonary involvement and severe disease (116). When combined with neutrophil to lymphocyte ratio (NLR), the dramatic decrease in eosinophil levels has a higher predictive value and could help in COVID-19 diagnostic and risk stratification (117). Conversely, an increasing EC during COVID-19 disease is associated with a milder clinical course and better disease outcomes, including a lower incidence of complications and mortality (118).

EC have been included in several algorithms used to predict disease severity (119), such as the “COVID-19-REAL” risk stratification score used to identify patients who are likely to be presenting with COVID-19 (120) and the “PARIS” score, in which presenting EC < 60/µL were among several hematologic parameters used to predict the likelihood of a SARS-CoV-2 diagnosis (121). Of note, co-infection with SARS-CoV-2 and other viral respiratory pathogens including influenza and RSV is not rare and increases disease severity and mortality risk compared to SARS-CoV-2 mono-infection. Since early symptoms of COVID-19 overlap with these common conditions, several groups have explored the value of peripheral blood EC at patient presentation for distinguishing between COVID-19 and influenza virus infection. In addition, several algorithms have been developed to assist clinicians to discriminate between these two respiratory virus infections (122). Finally, although vaccine-associated aberrant inflammatory responses, including eosinophil accumulation in the respiratory tract, were observed in preclinical immunization studies targeting the related SARS-CoV and MERS-CoV pathogens, there are no reports on Th2-mediated pulmonary immunopathology associated with any of the currently used encapsulated mRNA-based COVID-19 vaccines (112). However, concern might be heightened when these vaccines become available to young children (123). A summary of the principal findings on the roles of eosinophils in respiratory viral infections is illustrated in **Table 1**.

**Table 1:** Role of eosinophils in respiratory viral infections.

Virus	Model	Major findings	Mechanism of action	References
**RSV**	**Human:** *In vitro*: peripheral blood eosinophils.	RSV can infect human eosinophils. Eosinophils internalize and inactivate RSV, and are activated by the virus.	Release of IL-6, IL-1α, IL-13, IL-15, G-CSF, and GM-CSF. Upregulation of CD69 and CD11b expression.	(66) (104) (59)
	**Mouse**: *In vitro*: bone marrow-derived eosinophils. *In vivo*: Intranasal injection of RSV in mice.	↓ Viral load; ↑ Eosinophils accumulation; ↑ CD11b; ECP release; production of NO; antiviral immunity.	TLR-7, CD11b, ECP, NO. Antiviral immunity through NO production, clearance of RSV via MyD88-dependent pathways, reduction of RSV-induced mucus hypersecretion.	(59) 85)
**IAV**	**Mouse:** *In vitro:* peripheral blood and bone marrow-derived eosinophils *In vivo*: Transfer of eosinophils from the lungs of allergen-sensitized and challenged mice into IAV-infected mice.	Acute asthma infected mice: ↑ numbers of eosinophils in the airways, faster virus clearance, ↑ CD8^+^ T cells and lower epithelial damage. Adoptive transfer of eosinophils: ↓ viral burden and ↑ CD8^+^ T cell numbers in the airways of recipient mice.	*In vitro*: Piecemeal degranulation, NO release, ↑ MHC-I and CD86, induction of CD8^+^ T cell responses by virus pulsed eosinophils.	(95) (76)
**PIV**	**Human:** *In vitro*: IFN-γ preincubated human eosinophils.	Eosinophils significantly decreased PIV titers.	TLR-MyD88 pathway. *In vitro*: NO generation through TLR-7, PIV human eosinophils infection is abortive.	(101)
	**Mouse:** IL5 transgenic mice, PIV infection, anti-IL5 antibodies treatment. **Guinea pig:** sensitization to a non-viral antigen, infection, anti-IL5 antibodies.	↑ Eosinophils in the lungs; ↓ viral content in the lungs; sensitization to a non-viral antigen leads to an eosinophil-mediated ↓ viral content in the lungs.	TLR-7 involvement and NO production	(100) (99)
**HRV**	**Human:** *In vitro*: peripheral blood eosinophils.	Eosinophils as APCs: induction of CD4^+^ T-cell proliferation and IFN-γ production.	Viral binding through ICAM-1, cooperation between T cells and eosinophils: T cells secreted IFN-γ leads to ↑expression of TLR-7 and TLR-8 on eosinophils.	(103)
	**Human:** anti-IL5 antibody treatment, HRV infection.	Eosinophils depletion leads to increased viral loads in nasal swabs.	↑ CD69 expression	(104) (105)
**SARS-CoV-2**	**Human**	↓ Eosinophils absolute count (EC) associated with higher mortality.	Unclear, likely multifactorial.	(18) (114) (110) (36)
		↑ EC correlate with immune recovery ↑ EC correlate with milder clinical course and better disease outcomes	Th2-specific pathways Lower level of C-reactive protein, role in mitigating the severity of inflammatory response.	(117) (113)
		Protective role of eosinophils against severe COVID-19 illness even if associated to allergic asthma.	Possible protective mechanisms of asthma and type 2 inflammation on COVID-19 infection, expression of SARS-CoV-2 entry receptors, antiviral activity of eosinophils and cross-reactive T-cell epitopes.	(111) (109) (124)
		Subset of eosinophils related to clinical deterioration Immune exhaustion of eosinophils and inhibition of Th2-mediated immune response	IFN-γ-mediated upregulation of CD62L on eosinophils precedes lung hyperinflammation. ↑ expression of the programmed death receptor ligand 1 (PD-L1) checkpoint and ↓ expression of CRTH2 (CD294).	(124) (125)
		Algorithms using eosinophil counts to predict disease severity and to make a differential diagnosis.	“COVID-19-REAL” risk stratification score used to identify patients who are likely to be presenting with COVID-19 “PARIS” score categorizes the pre-test probability of SARS-CoV-2 infection (eosinophil counts < 60 / µL) Blood eosinophil counts (< 0.01 × 10^9^/L) distinguishes between COVID-19 and influenza virus infection.	(119) (120) (121)
		Dysregulation of immune responses in Long-COVID patients.	Persistently activated eosinophils **↓** counts, activation and hyper-responsiveness up to 6 months after active disease	(126) (127)

Arrow up, increase; Arrow down, decrease.

### 5.2 Long COVID and eosinophils

Individuals infected with SARS-CoV-2 often experience severe respiratory complications and other prolonged symptoms post-infection (sequelae), referred to as “Long-COVID” or “Post-Acute Sequelae of COVID-19” (PASC) (128). According to the World Health Organization, Long-COVID is defined as the continuation or development of new symptoms 3 months after the initial SARS-CoV-2 infection, and occurs in at least 10% of SARS-CoV-2 infections (129). Furthermore, PASC is defined by the persistence of disease greater than 28 days following the onset of symptoms (128). Symptoms include fatigue, dyspnea, arthralgia, myalgia, heart palpitations, and memory issues sometimes affecting between 30% and 75% of recovering COVID-19 patients (130).

So far, little is known about the etiology of chronic sequelae following acute SARS-CoV-2 infection, although several hypotheses have been suggested, including persisting reservoirs of SARS-CoV-2 in tissues, immune dysregulation, autoimmunity and molecular mimicry (126, 129). As described above, an increased EC during COVID-19 disease is associated with a milder clinical course and better disease outcomes, and, conversely, the extent of eosinopenia is found to be a marker for severe disease. However, there are few data in the literature indicating the role of eosinophils in long COVID. Longitudinal studies have revealed sustained dysregulation of immune responses in PASC, involving a reduction in naïve T- and B-cells, a decrease in the numbers of conventional dendritic cells, highly activated myeloid cells and T cells, elevated pro-inflammatory cytokine levels as well as persistently activated monocytes, mast cells, and eosinophils (109, 127, 129, 131).

A comprehensive study, comparing patients with acute COVID-19 disease and 3 to 6 months after active disease, showed specific neutrophil and eosinophil activation patterns, suggesting that the neutrophil and eosinophil compartments are long-term affected by COVID-19 and may be involved in the pathogenesis of long COVID (132). In particular, although blood EC were lower during the acute infection than 3 to 6 months after COVID-19, their numbers did not fully normalize, and activation and hyper-responsiveness persisted in the eosinophilic compartment up to 6 months after active disease (132). Another study found that patients in the acute COVID-19 phase presented with eosinophilia, and EC significantly increased up to 90 days of long COVID. Subsequently, eosinophils counts decreased to basal levels after 3 months, without any other changes after 6 and 12 months of observation (133).

Although patients with severe asthma are at an increased risk of developing long COVID a protective role for eosinophils and type 2 cytokines has been hypothesized. In a study assessing the long COVID outcomes after 6 to 12 months of an asthma population, authors demonstrated that eosinophilic and type 2-asthma could protect against complications of prolonged COVID, as compared to patients with severe asthma who presented a worse prognosis (134).

It has also been reported that levels of circulating granulocyte populations, including eosinophils, were not significantly different among participants with long COVID relative to matched control groups (135). Future studies must account for the long-term effects of COVID-19 on granulocyte populations, in terms of different counts and immune activation, to identify markers for patients at risk for developing a more severe presentation of long COVID, and for enabling better management of this condition”.

### 5.3 Mechanisms underlying eosinopenia and eosinophil responses to COVID-19

The precise mechanisms underlying eosinopenia associated with COVID-19 remain unclear at this time. Eosinopenia may result from one or a combination of factors, including decreased production and/or release of eosinophils from the bone marrow, increased sequestration within the vasculature (i.e., margination), increased migration to somatic tissues, and/or decreased survival in peripheral circulation. Nevertheless, the precise mechanisms underlying eosinopenia associated with COVID-19 remain unclear at this time.

Whether eosinophils may have an antiviral or deleterious role in the immune response against SARS-CoV-2 infection is still an open question (114). A number of clinical and pre-clinical studies have suggested a role for eosinophils in antiviral immunity and protection against the development of the uncontrolled inflammatory response underlying the severe COVID-19 disease (136). For example, eosinophilia in symptomatic COVID-19 patients has been linked to a lower level of inflammatory markers such as high-sensitivity CRP, suggesting a protective role of eosinophils in mitigating the severity of inflammatory diseases through an inhibitory mechanism (114). Finally, as stated before, eosinophils express a broad range of TLRs, such as TLR7, which has been shown to enable eosinophils to recognize single-stranded RNA viruses including coronaviruses (62).

## 6 IL-33 and eosinophils in respiratory viral infections and asthma co-morbidity

Epithelial-derived cytokines including IL-33, IL-25, and thymic stromal lymphopoietin (TSLP) play an important role in the development of viral-induced airway inflammation (137). IL-33 is an alarmin released by epithelial cells upon stress or injury induced by different environmental stimuli, such as airborne allergens and viruses. At the pulmonary level, exposure to exogenous or endogenous (i.e., epithelial-derived) IL-33 promotes the recruitment of eosinophils *via* stimulation of group 2 innate lymphoid cells (ILC2). Moreover, eosinophils respond directly to IL-33 resulting in increased expression of activation markers (i.e., CD69, CD11b), degranulation and survival (138).

IL-33 can stimulate both Th1 and Th2-types of immune responses in virtue of the pleiotropic expression of its specific receptor ST2 by virtually all hematopoietic cells. In mice, intranasal infection with RSV induced both the production of IL-33 and the expression of ST2, which was accompanied with a massive infiltration of ST2^+^CD45^+^ cells in the lungs, suggesting that during the early phase of RSV infection, IL-33 targeting of ST2 expressing cells may play a critical role for the development of RSV-induced airway inflammation. Blocking ST2 signaling diminished RSV-induced eosinophil recruitment and Th2-associated cytokines in the lungs of infected mice but did not affect the production of Th1-type cytokines nor pulmonary viral growth and clearance. These results indicate that IL-33/ST2 signaling is involved in RSV-induced, Th2-associated airway inflammation but not in protective immunity (139, 140).

The involvement of IL-33/ST2 axis in airway inflammation was also demonstrated in human rhinovirus (RV) infection model. In RV-infected mice, ST2 deficiency significantly reduced the levels of proinflammatory cytokines, including IL-33, and neutrophil mediated airway inflammation. By contrast, ST2 expression was associated with increased viral loads in the BAL of mice and in human epithelial cells infected *in vitro*. These data suggest that ST2 promotes proinflammatory responses to RV infection and increasing of airway infection (141). Of note, IL-33 and eosinophils enhance RV-induced airway inflammation and suppress IFN-β or IFN-λ expression and antiviral immunity (137). In particular, eosinophils directly suppress RV-induced type I IFN production by plasmacytoid dendritic cells (pDC) (142) and by epithelial cells (143) *via* release of TGF-β. Accordingly, patients with eosinophilic asthma displayed decreased levels of IFN-β (144).

**Figure 2:**
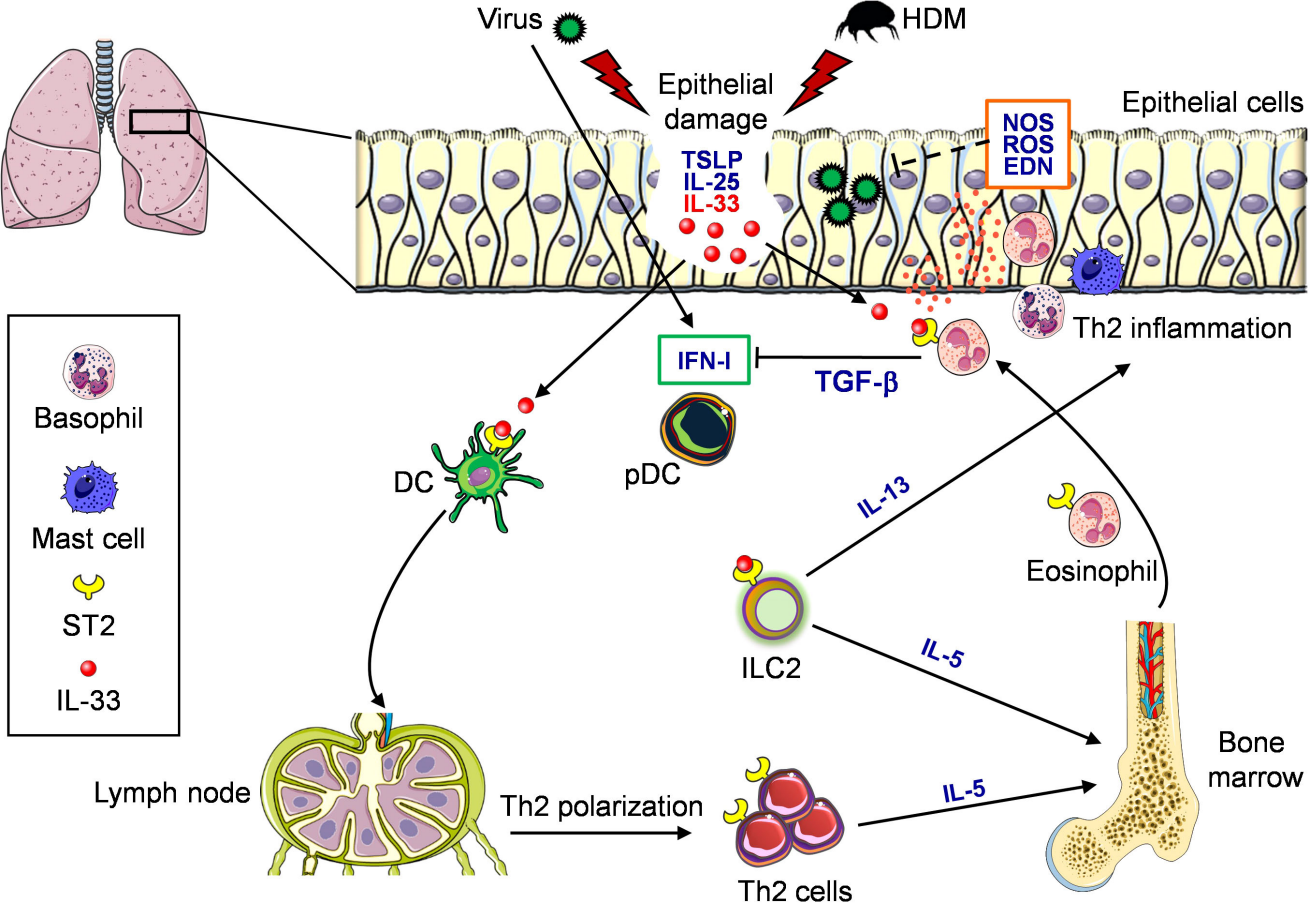
Role of eosinophils and IL-33/ST2 in the context of virus and allergen exposure in the airways. Virus infection and allergen (e.g., HDM) exposure induce damage in the lung epithelium. Both events result in the release of epithelial-derived alarmins, namely TSLP, IL-25 and IL-33. In particular, IL-33 binds to its specific receptor ST2 expressed by many immune cell types present in the lung. DC respond to IL-33 and migrate to draining lymph node where they prime naive Th cells inducing polarization of Th2 cells, which subsequently migrate to the lung and release IL-5. ST2-expressing ILC2 cells respond to IL-33 producing IL-5 and IL-13. IL-5 stimulates the differentiation of eosinophils from bone marrow. Eosinophils then migrate to the lung where they respond to IL-33, resulting in degranulation (orange dots) and release of different substances. These eosinophils, on the one hand, support Th2 inflammation sustained by ST2-derived IL-13 and recruitment of basophils and mast cells and, on the other hand, may prevent virus replication through the release of ribonucleases, reactive oxygen species (ROS) and nitric oxide synthase (NOS). Conversely, eosinophils may reduce virus-induced type I IFN (IFN-I) production by pDC through release of TGF-β.

Respiratory viral infections often require hospitalizations in asthmatic individuals, and airway-secreted cytokines, particularly IL-33, contribute to allergic exacerbations by amplifying type 2 inflammation. Viral infections caused by RSV in children (145) and RV in adults (146), respectively, are among the major drivers of asthma exacerbations. Many groups have attempted to identify mechanisms of the underlying virus-induced asthma exacerbation. In naïve mice, RV infection leads to neutrophilic lung inflammatory response with no recruitment of pulmonary eosinophils. However, in mice that had been previously sensitized with house dust mite (HDM) allergen, exposure to RV led to eosinophilia and elevated expression of several inflammatory factors associated with type 2 immunity, namely CCL17, CXCL1, CCL2, IL-33, and IL-13. Thus, previous allergen exposure skews antiviral response toward type 2 immunity and leads to allergic-like symptoms and overall exacerbated lung inflammation (147). Furthermore, in a model of OVA-allergic mice infected with RSV, neutralization of IL-33 significantly reduced ILC2, eosinophils, and the prototypical allergic proteins IL-5, IL-13, CCL17 and CCL22, further indicating the key role played by IL-33 in RSV-induced asthma exacerbation (148). The possible roles of IL-33 and eosinophils during concomitant allergen exposure and respiratory virus infection are summarized in **Figure 2**.

A recent study showed that higher expression of Th2-related genes and lower expression of type I IFN-related genes in upper airways cells of asthmatic children with RV infection were associated with a shorter time to exacerbation (149). Interestingly Altman and co-Workers identified by scRNAsec analysis of collected nasal cells a gene core associated with IL-33 and epithelial cell repair. However, one caveat in this study is that nasal epithelial cells were used as a proxy for lower-airways cells, thus not fully recapitulating the lung mucosal environment (150). A number of studies have demonstrated that lower levels of type I IFNs are produced by bronchial epithelial cells (146, 151, 152) and pDC (153) from asthmatic patients, compared to non-asthmatics, during RV or RSV infections. The suppression of innate antiviral response allows for virus spread and results in tissue damage, thus contributing to asthma exacerbation.

## 7 Eosinophils, COVID-19 and allergic asthma

Different from RV and RSV, pre-existing eosinophil-associated disorders (e.g., asthma, eosinophilic gastrointestinal disorders and allergic diseases) do not represent a relevant risk factor for COVID-19 susceptibility nor a predictor of the worst clinical course of disease (154). In fact, several recent studies suggest that a diagnosis of asthma may be associated with some degree of protection (155) with a lower hospitalization risk when compared with non-allergic asthma (156). Interestingly asthma patients with pre-existing eosinophilia (absolute eosinophils count, AEC >/= 150 cell/µl) had lower risk for COVID-19 admission, and asthma patients with eosinophilia during hospitalization due to COVID-19 had lower mortality compared with those whose AEC remained < 150 cells/µl (157). The reported evidences suggest that eosinophils might have a protective role against severe COVID-19 illness even if associated to allergic asthma (158).

The real contribution of eosinophils to the overall risk may also depend on the presence of environmental and behavioral factors (i.e., smoking), type and severity of asthma (i.e., non-type 2 asthma phenotypes), adherence to therapy, and comorbidities (159). The antiviral activity of eosinophils may partly contribute to the lower prevalence of allergic asthma in COVID-19 (160). In asthma patients, eosinophil activation is likely a double-edged sword causing both acute exacerbation of asthma and protection against serious outcomes of viral infection such as SARS-CoV-2 (158). This raises the opportunity to investigate the underlying mechanisms of the interaction between an allergic background and SARS-CoV-2 infection.

Possible protective mechanisms of asthma and type 2 inflammation on COVID-19 infection, such as the expression of SARS-CoV-2 entry receptors, antiviral activity of eosinophils and cross-reactive T-cell epitopes, have been reported (124). Currently available asthma treatments such as inhaled and oral corticosteroids, short- and long-acting β2 agonists, leukotriene receptor antagonists and biologicals have an impact on the outcome of COVID-19 patients. It has been proposed that inhaled corticosteroids may confer some degree of protection against SARS-CoV-2 infection and the development of severe disease by reducing the expression of angiotensin-converting enzyme-2 (ACE-2) and transmembrane protease serine in the lung. On the other hand, other biologicals used in severe asthmatic patients, namely IL-5 antagonists, anti-immunoglobulin E (anti-IgE), and anti-IL-4/IL-13 are able to modulate, decrease or deplete circulating eosinophils, and thus a detrimental effect in COVID-19 disease could be expected (159).

Rodriguez and co-Workers identified a unique subset of IFN-induced CD62L (L-selectin)-positive eosinophils that emerged just before clinical deterioration and lung hyperinflammation (125). CD62L expression is a previously reported marker of lung eosinophils (38) and it is possible that the IFN-γ-mediated upregulation of this marker on eosinophils leads to the influx of these cells into the lung tissue. These results are somewhat unexpected, as proinflammatory activation typically results in CD62L downregulation in eosinophils. The clinical consequences of this immunomodulatory response have not yet been defined. Similarly, Vitte and co-Workers (161) described typical a COVID-19 signature affecting first-line immune cells (neutrophils, eosinophils, and basophils) characterized by immune exhaustion evidenced by increased expression of the programmed death receptor ligand 1 (PD-L1) checkpoint in eosinophils and basophils, decreased expression of integrin CD11b, and Th2-related CRTH2 and increased counts of CD15^+^CD16^+^ neutrophils, correlating positively with disease severity (38). These data suggest that phenotypic markers of circulating granulocytes are strong discriminators between infected and uninfected individuals as well as between severity stages.

Similarly to other known respiratory virus infections, exposure to SARS-CoV-2 induces the expression of IL-33, correlating with T-cell activation and lung disease severity (162). Although IL-33 may participate in the pathogenesis of COVID-19 (163) this cytokine has also been reported to promote antiviral cytotoxic T cell responses and higher antibody production (164). Stanczak and co-Workers reported that after recovery from COVID-19, individuals have persisting, circulating peripheral blood mononuclear cells (PBMCs) that produce IL-33 in response to virus-specific T cell activation, which correlates with seropositivity (165). This finding suggests that persistent production of IL-33 in COVID-19 convalescent individuals may confer an advantage in the case of secondary exposure.

## 8 Currently used and novel tools for the study of eosinophils.

**Figure 3:**
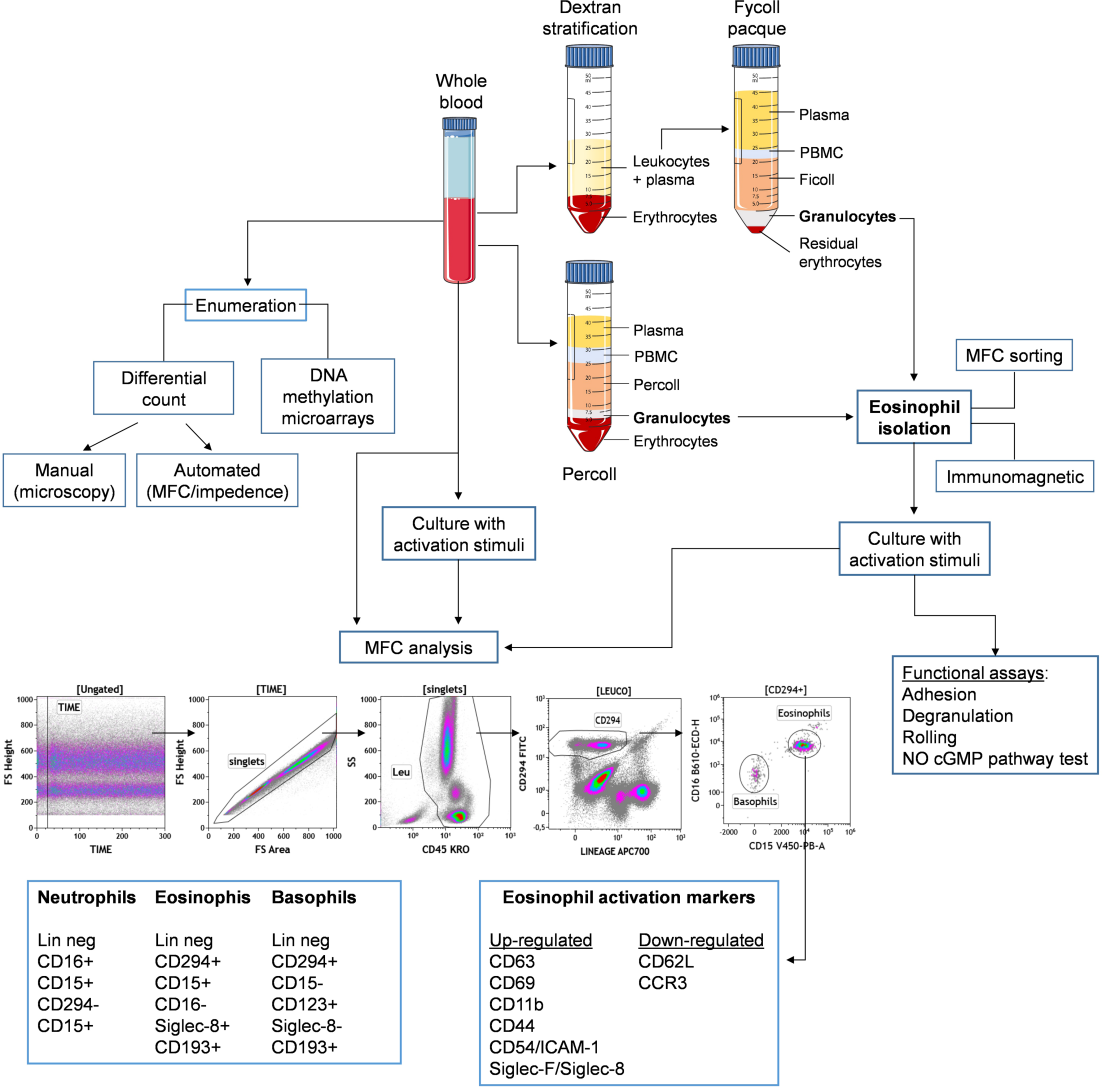
Schematic representation for principal methods of isolation and study of eosinophils from peripheral blood. Whole blood samples can be either directely stimulated in culture and stained with a multicolor panel to distinguish eosinophils from other leukocytes, such as neutrophils and basophils, based on indicated surface markers. Alternatively, eosinophils can be isolated from whole blood by density gradient enrichment followed by immunomagnetic or cell sorting purification. Eosinophils can be then assessed for phenotype (by expression of indicated activation markers) and function, either *ex vivo* or following *in vitro* stimulation.

Eosinophils can be retrieved in many biological districts, ranging from peripheral blood to many tissues (mainly mucosal) and fluids, such as bone marrow (166), sputum, bronchoalveolar lavage (167), urine (168), tears (169), breast milk (170), cervicovaginal (171) as well as cerebrospinal fluid (172). Tissue resident eosinophils are usually studied by means of microscopy tools (IHC, IF, EM). Blood eosinophils can be characterized by both enumeration and functional characterization, represented by activation, degranulation, cytotoxic and cytokine production. In this section, we will overview current and innovative methods, models and tools that have contributed to the advances in the dissection of various eosinophil subsets and their roles in the peripheral blood (summarized in **Figure 3**).

### 8.1 Enumeration and isolation

Paul Ehrlich published the methods for staining blood films and for differential blood cell counting using coal tar dyes and mentioned the eosinophils for the first time in 1879. Eosin is a bright red synthetic dye that stains basic proteins due to its acidic nature (173). The simplest test to enumerate blood eosinophils is the whole and differential blood cell count. Leukocytes can be counted manually in Neubauer chambers or with automated counters. To differentially count leukocyte subsets, a drop of blood is thinly spread over a glass slide, air dried, and stained with May-Grunewald-Giemsa technique, based on methylene blue and eosin. Count is performed by visual examination of blood smear by expert operators (174) and white cells are classified into lymphocytes, monocytes and granulocytes, the latter distinguished in neutrophils, eosinophils and basophils, named according to their characteristic staining: basophils stain dark blue, eosinophils red, and neutrophils stain pink. Machines have been developed to perform automated differential counts using multiple parameters and methods (such as fluorescence flow cytometry and impedance) (175–177). However, automated methods are less sensitive at identifying abnormal or immature cells (178). Novel multicolor flow cytometry approaches are also investigated to overcome inconsistency between manual and automated hematology analyzer count (179, 180).

Eosinophils can be enriched from peripheral blood by Percoll density gradient method (181) or by Dextran stratification followed by density centrifugation in Ficoll-Paque (182). Isolation of >90% pure eosinophils can be achieved by immuno-magnetic (183) or flow cytometry-based cell sorting (184). Since excessive manipulation may induce eosinophil activation, negative sorting protocols, which yield untouched cells, should be recommended.

### 8.2 Eosinophil characterization by flow cytometry

Unstained human eosinophils exhibit unusually bright autofluorescence, which could make flow cytometry analysis of eosinophils somewhat difficult, requiring careful evaluation of actual positive staining when using fluorochrome-labelled monoclonal antibodies. Conversely, this extraordinarily bright autofluorescence pattern represents an instrumental tool for easy identification of eosinophils, which allows to either include or exclude them from flow cytometry or fluorescence microscopy analysis. Indeed, eosinophil fluorescence is associated with the cytoplasmic granules of the cells. Eosinophil granule extracts, containing an as-yet-undefined eosinophil fluorescence factor, exhibited excitation maxima at 370 nm and 450 nm, with maximum emission at 520 nm. Eosinophils adhering to opsonized parasites *in vitro* deposit fluorescent material onto the parasite surface. Eosinophil fluorescence was of sufficient intensity to allow the preparation of viable, highly enriched (greater than or equal to 98%), eosinophil suspensions from peripheral blood of normal and eosinophilic donors using a fluorescence- activated cell sorter. Quantitative studies of eosinophil autofluorescence were performed using flow microfluorometry. Fluorescence intensity of blood eosinophils from normal volunteers and eosinophilic patients varied inversely with the log of the donor’s absolute eosinophil count regardless of clinical diagnosis (185).

In blood and other biological fluids, multiparameter flow cytometry (MFC) enables fast high-throughput profiling and classification of granulocyte subsets: neutrophils, basophils and eosinophils (186) and further differentiation of eosinophil subsets (187–190). Eosinophils express a variety of cell surface receptors relevant to their identity, maturation, activation, apoptosis, adhesion and rolling, homing, migration in tissues as well as their interaction with chemokines and cytokines, as illustrated in **Table 2** (191, 192).

**Table 2:** Major eosinophil receptors.

**Cytokine receptors**	**Pattern-recognition receptors (PRRs)**	**Chemokine receptors**
IL-2R	TLR1	CCR3
IL-3R (CD123)	TLR5	CCR1
IL-4R	TLR7	CCR2
IL-5R (CD125)	TLR8	CXCR3
IL-9R	TLR9	CXCR4
IL-10R	TLR2	CCR4
IL-13R	TLR3	CCR5
IL-17R	TLR4	CCR6
IL-23R	TLR6	CCR8
IL-27R	TLR10	CCR9
IL-31R	NOD1	CXCR2
IL-33R	NOD2	FPR1
TSLPR	RIGs	
GM-CSFR (CD116)	RAGE	**Complement receptors**
c-kit (CD117)		CR1 (CD35)
IFN-γR	**Immunoglobulin receptors**	CR3bi (Mac1; CD11b–CD18)
TGF-βR	FCaR (CD89)	CD88 (C5aR)
CD131	FCgRII (CD32)	C3aR
IL-1R	FcϵRI*	
TNF-αR	FcϵRII	**Inhibitory receptors**
		FCgRIIB (CD32)
**Adhesion molecules**	**Lipid mediator receptors**	LIR3 (CD85a)
CD62L (L-selectin)	DP2 prostaglandin receptor (CRTH2)	KIR2DL3
CD162 (PSGL1)	CysLT1R	CD300a
CD15 (Sialil Lewis X)	CysLT2R	Siglec-8
CD34	PAFR	Siglec-10
CD44	LTB4R	
CD54 (ICAM-1)	DP1 prostaglandin receptor	**Other receptors**
CR3bi (Mac1; CD11b–CD18)	EP2 prostaglandin receptor	CD52
CR4 (CD11c–CD18)		Histamine 4R
LFA-1 (CD11a–CD18)	**Proteinase-activated receptors**	CD95
VLA4 (CD49d–CD29)	PAR1	CD69
2B4 (CD244)	PAR2	Paired immunoglobulin-like receptor B (PIRB)
CD43 (lukosialin)		MHCII
ESL-1	**Costimulatory receptors**	PPAR γ
	CD28	
	CD86	

*Present on human eosinophils, but not in mouse eosinophils.

Blood eosinophils can be defined as leukocytes (CD45 positive), showing high side scatter and high auto-fluorescence, expressing both CD294 and CD15, lacking CD3 (T cell marker), CD19 (B cell marker), CD56 (NK cell marker), CD14 (monocyte marker) and CD16 (FcγRIII receptor). In addition, human blood eosinophils are reported to express high levels of CC-chemokine receptor-3 (CCR3) which are highly expressed both on circulating basophils and eosinophils where it is responsible for both migration and degranulation (193–195).

CD294, also known as CRTH2, is a seven-transmembrane G-protein-coupled receptor known as the chemoattractant receptor-homologous molecule expressed on Th2 cells, while CD15, also known as sialyl Lewis x, is involved in leukocyte rolling and missing in basophils (196). CRTH2 binds to the ligand prostaglandin D2 (PGD2), constitutively expressed on circulating basophils (197)

Eosinophils can be distinguished from neutrophils by the lack of CD16 expression and by the presence of CD49d (a costimulatory receptor) (186). Another strategy is based on exclusion of CD14 and CD16 and inclusion of the very specific marker Siglec-8, a late differentiation marker (198), and CD66b among granulocytes defined by high sideward scatter (199, 200). CD66b is a typical activation marker for human granulocytes, but its biological function is unknown in eosinophils. It was found that CD66b is highly expressed on the surface of eosinophils isolated from healthy individuals. Engagement of CD66b, but not CD66a, activated a Src kinase family molecule, hemopoietic cell kinase (Hck), and induced cellular adhesion, superoxide production, and degranulation of eosinophils. Importantly, CD66b was constitutively and physically associated with the beta2 integrin, CD11b (201). Binding of exogenous or endogenous carbohydrate ligands(s) to CD66b may be important in the release of proinflammatory mediators by human eosinophils (202).

Regarding maturation determinants, eosinophilic promyelocytes are CD11b^–^ and CD62L^–^, eosinophilic myelocytes are CD11b^+^ and CD62L^–^, eosinophilic metamyelocytes are CD11b^+^ and CD62L^dim^ and mature eosinophils are CD11b^+^ and CD62L^+^. CD62L, an adhesion molecule present in multiple blood cells, is shed from the eosinophil membrane after passage through the endothelium and its expression, coupled to other markers, can differentiate inflammatory CD62L^lo^ (iEOS) from resident CD62L^+^ eosinophils (rEOS) (38, 203).

### 8.3 Eosinophil activation

Several biologically relevant eosinophil-surface proteins have been proposed to assess eosinophil activation, including toll-like receptors, Fc receptors, gangliosides and glycoproteins: CD69, CD63, L-selectin (CD62L), intercellular adhesion molecule-1 (ICAM-1, CD54), CD44, P-selectin glycoprotein ligand-1 (PSGL-1, CD162), cytokine receptors, integrins including αM integrin (CD11b), and activated conformations of Fc receptors and integrins. Upon activation, some of these surface proteins are decreased or shed, such as CD23, CD31 and PSGL-1 (CD162) (204) while the others, including CD35, CD11b, CD66, CD69 and CD81, are increased (205).

The most consistent and readily detectable activation marker is the almost universal leukocyte early activation marker CD69, since it is not normally expressed on the surface of eosinophils but it can be up-regulated on eosinophil membrane. Conversely, alteration of other phenotypic markers is more of a subtle change in the level of surface expression rather than presence versus absence. The surface and intracellular distribution of CD69 was previously investigated with a whole-blood cell-membrane permeabilization technique, the FOG method, and flow cytometry (206). Eosinophils and neutrophils from healthy donors have a preformed intracellular pool of CD69, which is mobilized on the cell surface on eosinophils, but not on neutrophils, to various extents by selected stimuli.

Previous studies demonstrate the dynamic nature of eosinophil surface molecules and the important role for whole-blood staining in developing and understanding the role of eosinophils in inflammatory reactions (207). In fact, simultaneous comparison of purified eosinophils and whole-blood cells revealed significant differences in the levels of expression of various surface molecules, suggesting that the purification process may activate the eosinophils (208). For example, IL-33-induced activation of human (209) and mouse (138) eosinophils results in increased expression of CD69 and CD11b, the latter promoting adhesion and contact-dependent degranulation. It was also demonstrated that eosinophils can kill target cells *in situ* by protease-induced apoptosis mediated by their production of granzyme B, perforin, or cationic proteins, such as EPO and ECP, as revealed by intracellular MFC or confocal microscopy (138, 210).

Focusing on the topic of the present review, a number of markers expressed by eosinophils are involved in the recognition and orchestration of antiviral responses to respiratory viruses (66, 78, 102), including the coronavirus receptors CD13 and CD147, the measles virus receptor CD46, and the Echo-/Coxackie virus receptor CD55. Moreover, once activated by cytokines, such as IFN-γ or TNF-α, eosinophils may display additional virus receptors, such as the rhinovirus receptor CD54 (ICAM-1) (211). Furthermore, distinct immunotypes were evident in COVID-19 patients, with altered expression of several receptors involved in activation, adhesion, and migration of granulocytes (e.g., CD62L, CD11a/b, CD69, CD63, CXCR4). A comprehensive granulocyte characterization in COVID-19, which reveals specific immunotypes with potential predictive value for key clinical features associated with COVID-19, has been described (212).

### 8.4 Eosinophil characterization needs MFC harmonized assays

Low abundance, activation status, sample origin and relatively low life span of blood eosinophils are challenging factors for a reliable characterization of these granulocytes by MFC (213). MFC is a powerful technology but, given the complexity of polychromatic assays currently used, a harmonization process is crucial to guarantee reproducibility of data among laboratories, especially in multicenter trials (214, 215).

Commercial MFC panels have been designed to identify eosinophils as well as other granulocytes by means of dried/lyophilized antibody mixtures. Lyophilized reagents have already proven to yield high reproducibility and efficient standardization in large‐scale projects, such as the ONE study (216) and the PreciseADS study (217). These panels can be implemented in order not only to quantify the proportion of eosinophils in the blood but also to assess their activation status by means of markers, such as CD62L, CD11b or CD69.

Based on the multiple surface molecules expressed by eosinophils (**Table 2**) (19, 218), several panels can be designed *ad hoc* depending on research context. As examples, in the contest of allergy, several panels have been described based on CD40 and ICOS ligand, applied to study chronic rhinosinusitis (219), Siglec-8, CD62L, CD125, CD101 and CD123 expression in asthma and COPD patients (220) or CD63, CD193 (CCR3), CD294 (CRTH2) and HLA-DR in the blood of asthma patients (221).

### 8.5 DNA methylation microarrays

An innovative method based on DNA methylation microarrays can be employed to examine cell-type composition in complex tissues. An expanded version of a reference-based deconvolution of blood DNA methylation to include 12 leukocyte subtypes (neutrophils, eosinophils, basophils, monocytes, naïve and memory B cells, naïve and memory CD4^+^ and CD8^+^ T cells, natural killer, and T regulatory cells) is described by Salas and co-Workers. The method provides 56 immune profile variables comprising markers associated to eosinophil identity. These libraries enable a detailed representation of immune-cell profiles in blood using only DNA and facilitate their standardization, thorough investigation of immune profiles in human health and disease (222).

### 8.6 Degranulation and adhesion assay

A comprehensive and organized list of methods to assess degranulation in eosinophil is enclosed in a recent review (223). Among these, measuring the EPO amount in a cell-free fluid is a valuable method to indirectly determine liberated granules. EPO can be measured by a modified procedure described by Bozemann. Briefly, eosinophils are incubated in the presence of H_2_O_2_ and 1.4 mM tetramethybenzidine, 0.3 M sucrose and 3 mM decyltrimethylammonium bromide. During 3 min at room temperature, absorbance is measured at 650 nm in an ELISA plate reader in 30 sec intervals and the increase in absorbance is calculated (224). Alternatively, a sandwich ELISA employing pairs of EPO specific antibodies is commercially available.

Eosinophil adhesion to a particular cell correlates to its activation state but it also depends on the specific membrane molecules expression on target cells. Adhesion of eosinophils to a target cell can be quantitatively determined by flow cytometry, by labelling eosinophils and target cells with different fluorescent dyes (e.g., PKH26 and PKH67) before co-culture (1-2 hours). Determination of the percentage of cell conjugates by double positivity of the dyes, with respect to single dye positive eosinophils, can give a good estimate of the extent of eosinophil adhesion to target cells (138). Moreover, Grosicki and co-Workers described a “human eosinophils adhesion to endothelium assay” for drug screening based on co-culture of eosinophils and a human endothelial cells. After co-culture, adherent cells are stained with Hoechst 33342 for identification of eosinophils by nuclei morphological properties through fluorescence microscopy (225). Finally, the nitric oxide-cGMP pathway involved in leukocyte rolling, adhesion, and extravasation can be investigated in isolated eosinophils by commercial available kits (211).

## 9 Concluding remarks

Biomarkers are pivotal parameters for detecting the presence or absence of disease, monitoring changes in the clinical course of an illness, interpreting the response to an intervention or the environment, predicting treatment response, identifying populations at high risk for disease progression, recurrence, or clinical events, identify susceptibility or risk and to determine the likelihood of adverse events. Recent evidences highlight the importance of measuring blood eosinophils in respiratory diseases such asthma (226) and Chronic obstructive pulmonary disease (COPD) (227–230) for determination of disease severity and to aid in treatment decisions. In the context of respiratory viral infections, eosinophils have emerged as a valuable diagnostic and prognostic tool in the management of the SARS-CoV-2 infection (117). However, while eosinopenia seems to be a frequent feature in severe COVID-19, the precise role of eosinophils and the underlying mechanisms in this emerging disease are still poorly understood. Of note, allergic diseases and asthma do not aggravate the risk of severe/critical COVID-19 outcomes and patients with allergic disease should continue with standard treatment (112). Although detection of SARS-CoV-2 infection in allergic patients might be challenging due to overlap of allergy and COVID-19 symptoms, screening for SARS-CoV-2 is imperative to minimize viral transmission and to differentiate between COVID-19 symptoms and allergies. Further studies are required to fully understand the relationship between eosinophils and other respiratory viruses during certain conditions, such as asthma. Finally, standardized protocols for improving eosinophil characterization are desirable for the use of these granulocytes as biomarkers in the screening and management of respiratory diseases.

## Author contributions

IM, CAf and GS designed the manuscript. IM, VLS, FU, SM, CAn, CAf, GS collected data and drafted the manuscript. CAn and GS prepared the figures. FU, SM and VLS prepared the Tables. IM, VLS, SM FU, CAf, GS revised the manuscript. IM and GS edited the manuscript. All authors approved the manuscript.

## References

[1] Huber HL Koessler KK. The pathology of bronchial asthma. Arch Intern Med (1922) 30:689–760. doi: 10.1001/archinte.1922.00110120002001

[2] Long H Liao W Wang L Lu Q. A player and coordinator: the versatile roles of eosinophils in the immune system. Transfus Med Hemother (2016) 43:96–108. doi: 10.1159/000445215 PMC4872051 27226792

[3] Radonjic-Hösli S Simon HU. Eosinophils. Chem Immunol Allergy (2014) 100:193–204. doi: 10.1159/000358735 24925399

[4] Huang L Appleton JA. Eosinophils in helminth infection: defenders and dupes. Trends Parasitol (2016) 32:798–807. doi: 10.1016/j.pt.2016.05.004 PMC5048491 27262918

[5] Lambrecht BN Hammad H. The immunology of asthma. Nat Immunol (2015) 16:45–56. doi: 10.1038/ni.3049 25521684

[6] Marichal T Mesnil C Bureau F. Homeostatic eosinophils: characteristics and functions. Front Med (2017) 4:101. doi: 10.3389/fmed.2017.00101 PMC5504169 28744457

[7] Sastre B Rodrigo-Muñoz JM Garcia-Sanchez DA Cañas JA Del Pozo V. Eosinophils: old players in a new game. J Investig Allergol Clin Immunol (2018) 28:289–304. doi: 10.18176/jiaci.0295 30059011

[8] Wen T Rothenberg ME. The regulatory function of eosinophils. Microbiol Spectr (2016) 4:10. doi: 10.1128/microbiolspec.mchd-0020-2015 PMC5088784 27780017

[9] Constantine GM Klion AD. Recent advances in understanding the role of eosinophils. Fac Rev (2022) 11:26. doi: 10.12703/r/11-26 36225210 PMC9523544

[10] Chu VT Beller A Rausch S Strandmark J Zänker M Arbach O. Eosinophils promote generation and maintenance of immunoglobulin-a-expressing plasma cells and contribute to gut immune homeostasis. Immunity (2014) 40:582–93. doi: 10.1016/j.immuni.2014.02.014 24745334

[11] Lee EH Itan M Jang J Gu HJ Rozenberg P Mingler MK. Eosinophils support adipocyte maturation and promote glucose tolerance in obesity. Sci Rep (2018) 8:582–93. doi: 10.1038/s41598-018-28371-4 PMC6028436 29967467

[12] Goh YPS Henderson NC Heredia JE Eagle AR Odegaard JI Lehwald N. Eosinophils secrete IL-4 to facilitate liver regeneration. Proc Natl Acad Sci USA (2013) 110:9914–9. doi: 10.1073/pnas.1304046110 PMC3683773 23716700

[13] Liu J Yang C Liu T Deng Z Fang W Zhang X. Eosinophils improve cardiac function after myocardial infarction. Nat Commun (2020) 11:6396. doi: 10.1038/s41467-020-19297-5 33328477 PMC7745020

[14] Andreev D Liu M Kachler K Llerins Perez M Kirchner P Kölle J. Regulatory eosinophils induce the resolution of experimental arthritis and appear in remission state of human rheumatoid arthritis. Ann Rheum Dis (2021) 80:451–68. doi: 10.1136/annrheumdis-2020-218902 33148700

[15] Arnold IC Artola-Borán M Tallón de Lara P Kyburz A Taube C Ottemann K. Eosinophils suppress Th1 responses and restrict bacterially induced gastrointestinal inflammation. J Exp Med (2018) 215:2055–72. doi: 10.1084/jem.20172049 PMC6080907 29970473

[16] Huang R Xie L He J Dong H Liu T. Association between the peripheral blood eosinophil counts and covid-19 a meta-analysis. Med (United States) (2021) 100:e26047. doi: 10.1097/MD.0000000000026047 PMC8202592 34114990

[17] Lamichhane PP Samarasinghe AE. The role of innate leukocytes during influenza virus infection. J Immunol Res (2019) 2019:8028725. doi: 10.1155/2019/8028725 31612153 PMC6757286

[18] Lombardi C Berti A Cottini M. The emerging roles of eosinophils: implications for the targeted treatment of eosinophilic-associated inflammatory conditions. Curr Res Immunol (2022) 3:42–53. doi: 10.1016/j.crimmu.2022.03.002 35496822 PMC9040157

[19] Kanda A Yun Y Van Bui D Nguyen LM Kobayashi Y Suzuki K. The multiple functions and subpopulations of eosinophils in tissues under steady-state and pathological conditions. Allergol Int (2021) 70:9–18. doi: 10.1016/j.alit.2020.11.001 33243693

[20] Varricchi G Galdiero MR Loffredo S Lucarini V Marone G Mattei F. Eosinophils: the unsung heroes in cancer? Oncoimmunology (2018) 7:e1393134. doi: 10.1080/2162402X.2017.1393134 29308325 PMC5749653

[21] Ramirez GA Yacoub MR Ripa M Mannina D Cariddi A Saporiti N. Eosinophils from physiology to disease: a comprehensive review. BioMed Res Int (2018) 2018:9095275. doi: 10.1155/2018/9095275 29619379 PMC5829361

[22] Jackson DJ Akuthota P Roufosse F. Eosinophils and eosinophilic immune dysfunction in health and disease. Eur Respir Rev (2022) 31:210150. doi: 10.1183/16000617.0150-2021 35082127 PMC9489126

[23] Akuthota P Wang H Weller PF. Eosinophils as antigen-presenting cells in allergic upper airway disease. Curr Opin Allergy Clin Immunol (2010) 10:14–19. doi: 10.1097/ACI.0b013e328334f693 PMC2865844 19949323

[24] Padigel UM Hess JA Lee JJ Lok JB Nolan TJ Schad GA. Eosinophils act as antigen-presenting cells to induce immunity to strongyloides stercoralis in mice. J Infect Dis (2007) 196:1844–51. doi: 10.1086/522968 PMC3154724 18190266

[25] Hogan SP Rosenberg HF Moqbel R Phipps S Foster PS Lacy P. Eosinophils: biological properties and role in health and disease. Clin Exp Allergy. (2008) 709–50. doi: 10.1111/j.1365-2222.2008.02958.x. 18384431

[26] Shi H-Z. Eosinophils function as antigen-presenting cells. J Leukoc Biol (2004) 76:520–7. doi: 10.1189/jlb.0404228 15218055

[27] Rodrigo-muñoz JM Gil-martínez M Sastre B Del Pozo V. Emerging evidence for pleiotropism of eosinophils. Int J Mol Sci (2021) 22:7075. doi: 10.3390/ijms22137075 34209213 PMC8269185

[28] Kovalszki A Weller PF. Eosinophilia. Prim Care Clin Off Pract (2016) 43:607–17. doi: 10.1016/J.POP.2016.07.010 PMC5293177 27866580

[29] Lee JJ Jacobsen EA McGarry MP Schleimer RP Lee NA. Eosinophils in health and disease: the LIAR hypothesis. Clin Exp Allergy (2010) 40:573–5. doi: 10.1111/j.1365-2222.2010.03484.x PMC2951476 20447076

[30] Mishra A Hogan SP Lee JJ Foster PS Rothenberg ME. Fundamental signals that regulate eosinophil homing to the gastrointestinal tract. J Clin Invest (1999) 103:1719–27. doi: 10.1172/JCI6560 PMC408388 10377178

[31] Kim HJ Jung Y. The emerging role of eosinophils as multifunctional leukocytes in health and disease. Immune Netw (2020) 20:e24. doi: 10.4110/in.2020.20.e24 32655972 PMC7327148

[32] Weller PF Spencer LA. Functions of tissue-resident eosinophils. Nat Rev Immunol (2017) 17:746–60. doi: 10.1038/nri.2017.95 PMC5783317 28891557

[33] Hogan SP Rosenberg HF Moqbel R Phipps S Foster PS Lacy P. Eosinophils: biological properties and role in health and disease. In: Allergy and allergic diseases, 2nd ed. Wiley (2009). 1 p. 258–94. doi: 10.1002/9781444300918.ch12 18384431

[34] Davoine F Lacy P. Eosinophil cytokines, chemokines, and growth factors: emerging roles in immunity. Front Immunol (2014) 5:570. doi: 10.3389/fimmu.2014.00570 25426119 PMC4225839

[35] Spencer LA Szela CT Perez SAC Kirchhoffer CL Neves JS Radke AL. Human eosinophils constitutively express multiple Th1, Th2, and immunoregulatory cytokines that are secreted rapidly and differentially. J Leukoc Biol (2008) 85:117–23. doi: 10.1189/jlb.0108058 PMC2626765 18840671

[36] Rodrigo-Muñoz JM Sastre B Cañas JA Gil-Martínez M Redondo N Del Pozo V. Eosinophil response against classical and emerging respiratory viruses: covid-19. J Investig Allergol Clin Immunol (2021) 31:94–107. doi: 10.18176/jiaci.0624 32540792

[37] Abdala-Valencia H Coden ME Chiarella SE Jacobsen EA Bochner BS Lee JJ. Shaping eosinophil identity in the tissue contexts of development, homeostasis, and disease. J Leukoc Biol (2018) 104:95–108. doi: 10.1002/JLB.1MR1117-442RR PMC6013365 29656559

[38] Mesnil C Raulier S Paulissen G Xiao X Birrell MA Pirottin D. Lung-resident eosinophils represent a distinct regulatory eosinophil subset. J Clin Invest (2016) 126:3279–95. doi: 10.1172/JCI85664 PMC5004964 27548519

[39] Rosenberg HF Dyer KD Foster PS. Eosinophils: changing perspectives in health and disease. Nat Rev Immunol (2013) 13:9–22. doi: 10.1038/nri3341 PMC4357492 23154224

[40] Jung Y Wen T Mingler MK Caldwell JM Wang YH Chaplin DD. IL-1β in eosinophil-mediated small intestinal homeostasis and IgA production. Mucosal Immunol (2015) 8:930–42. doi: 10.1038/mi.2014.123 PMC4481137 25563499

[41] Woodruff PG Modrek B Choy DF Jia G Abbas AR Ellwanger A. T-Helper type 2-driven inflammation defines major subphenotypes of asthma. Am J Respir Crit Care Med (2009) 180:388–95. doi: 10.1164/RCCM.200903-0392OC PMC2742757 19483109

[42] Bentley AM Jacobson MR Cumberworth V Barkans JR Moqbel R Schwartz LB. Immunohistology of the nasal mucosa in seasonal allergic rhinitis: increases in activated eosinophils and epithelial mast cells. J Allergy Clin Immunol (1992) 89:877–83. doi: 10.1016/0091-6749(92)90444-7 1532808

[43] Lambrecht BN Hammad H Fahy JV. The cytokines of asthma. Immunity (2019) 50:975–91. doi: 10.1016/J.IMMUNI.2019.03.018 30995510

[44] Hammad H Lambrecht BN. The basic immunology of asthma. Cell (2021) 184:1469–85. doi: 10.1016/J.CELL.2021.02.016 33711259

[45] Muraro A Lemanske RF Hellings PW Akdis CA Bieber T Casale TB. Precision medicine in patients with allergic diseases: airway diseases and atopic dermatitis-PRACTALL document of the European academy of allergy and clinical immunology and the American academy of allergy, asthma & immunology. J Allergy Clin Immunol (2016) 137:1347–58. doi: 10.1016/J.JACI.2016.03.010 27155030

[46] Wardlaw AJ Dunnette S Gleich GJ Collins JV Kay AB. Eosinophils and mast cells in bronchoalveolar lavage in subjects with mild asthma. relationship to bronchial hyperreactivity. Am Rev Respir Dis (1988) 137:62–9. doi: 10.1164/ajrccm/137.1.62 2447813

[47] Chen R Smith SG Salter B El-Gammal A Oliveria JP Obminski C. Allergen-induced increases in sputum levels of group 2 innate lymphoid cells in subjects with asthma. Am J Respir Crit Care Med (2017) 196:700–12. doi: 10.1164/RCCM.201612-2427OC/SUPPL_FILE/DISCLOSURES.PDF 28422515

[48] Walsh GM. Eosinophil apoptosis and clearance in asthma. J Cell Death (2013) 6:17. doi: 10.4137/JCD.S10818 25278777 PMC4147767

[49] Anwar ARF Moqbel R Walsh GM Kay AB Wardlaw AJ. Adhesion to fibronectin prolongs eosinophil survival. J Exp Med (1993) 177:839–43. doi: 10.1084/JEM.177.3.839 PMC2190928 8436913

[50] Gleich GJ Flavahan NA Fujisawa T Vanhoutte PM. The eosinophil as a mediator of damage to respiratory epithelium: a model for bronchial hyperreactivity. J Allergy Clin Immunol (1988) 81:776–81. doi: 10.1016/0091-6749(88)90931-1 3286717

[51] Drake MG Lebold KM Roth-Carter QR Pincus AB Blum ED Proskocil BJ. Eosinophil and airway nerve interactions in asthma. J Leukoc Biol (2018) 104:61–7. doi: 10.1002/JLB.3MR1117-426R PMC6541210 29633324

[52] Borek-Dorosz A Grosicki M Dybas J Matuszyk E Rodewald M Meyer-Zedler T. Identification of inflammatory markers in eosinophilic cells of the immune system: fluorescence, raman and CARS imaging can recognize markers but differently. Cell Mol Life Sci (2022) 79:52. doi: 10.1007/s00018-021-04058-4 PMC8739296 34936035

[53] Humbles AA Lloyd CM McMillan SJ Friend DS Xanthou G McKenna EE. A critical role for eosinophils in allergic airways remodeling. Science (80-) (2004) 305:1776–9. doi: 10.1126/science.1100283 15375268

[54] Cañas JA Sastre B Rodrigo-Muñoz JM Fernández-Nieto M Barranco P Quirce S. Eosinophil-derived exosomes contribute to asthma remodelling by activating structural lung cells. Clin Exp Allergy (2018) 48:1173–85. doi: 10.1111/CEA.13122 29451337

[55] Gieseck RL Wilson MS Wynn TA. Type 2 immunity in tissue repair and fibrosis. Nat Rev Immunol (2018) 18:62–76. doi: 10.1038/NRI.2017.90 28853443

[56] Fattouh R Jordana M. TGF-β, eosinophils and IL-13 in allergic airway remodeling: a critical appraisal with therapeutic considerations. Inflammation Allergy - Drug Targets (2008) 7:224–36. doi: 10.2174/187152808786848388 19075788

[57] Gaur P Zaffran I George T Rahimli Alekberli F Ben-Zimra M Levi-Schaffer F. The regulatory role of eosinophils in viral, bacterial, and fungal infections. Clin Exp Immunol (2022) 209:72–82. doi: 10.1093/cei/uxac038 35467728 PMC9307229

[58] Mnsson A Fransson M Adner M Benson M Uddman R Björnsson S. TLR3 in human eosinophils: functional effects and decreased expression during allergic rhinitis. Int Arch Allergy Immunol (2010) 151:118–128. doi: 10.1159/000236001 19752565

[59] Phipps S En Lam C Mahalingam S Newhouse M Ramirez R Rosenberg HF. Eosinophils contribute to innate antiviral immunity and promote clearance of respiratory syncytial virus. Blood (2007) 110:1578–86. doi: 10.1182/blood-2007-01-071340 17495130

[60] Wong CK Cheung PFY Ip WK Lam CWK. Intracellular signaling mechanisms regulating toll-like receptor-mediated activation of eosinophils. Am J Respir Cell Mol Biol (2007) 37:85–96. doi: 10.1165/rcmb.2006-0457OC 17332440

[61] Diebold SS Kaisho T Hemmi H Akira S Reis E Sousa C. Innate antiviral responses by means of TLR7-mediated recognition of single-stranded RNA. Science (80-) (2004) 303:1529–31. doi: 10.1126/science.1093616 14976261

[62] Nagase H Okugawa S Ota Y Yamaguchi M Tomizawa H Matsushima K. Expression and function of toll-like receptors in eosinophils: activation by toll-like receptor 7 ligand. J Immunol (2003) 171:3977–82. doi: 10.4049/jimmunol.171.8.3977 14530316

[63] Loo YM Gale M. Immune signaling by RIG-i-like receptors. Immunity (2011) 34:680–392. doi: 10.1016/j.immuni.2011.05.003 PMC3177755 21616437

[64] Lotfi R Herzog GI DeMarco RA Beer-Stolz D Lee JJ Rubartelli A. Eosinophils oxidize damage-associated molecular pattern molecules derived from stressed cells. J Immunol (2009) 183:5023–31. doi: 10.4049/jimmunol.0900504 19794066

[65] Arikkatt J Ullah MA Short KR Zhang V Gan WJ Loh Z. RAGE deficiency predisposes mice to virus-induced paucigranulocytic asthma. Elife (2017) 6:e21199. doi: 10.7554/eLife.21199 28099113 PMC5243115

[66] Rimmelzwaan GF Baars MMJW de Lijster P Fouchier RAM Osterhaus ADME. Inhibition of influenza virus replication by nitric oxide. J Virol (1999) 73:8880. doi: 10.1128/JVI.73.10.8880-8883.1999 10482647 PMC112914

[67] Dyer KD Percopo CM Fischer ER Gabryszewski SJ Rosenberg HF. Pneumoviruses infect eosinophils and elicit MyD88-dependent release of chemoattractant cytokines and interleukin-6. Blood (2009) 114:2649–56. doi: 10.1182/blood-2009-01-199497 PMC2756124 19652202

[68] Gleich GJ Loegering DA Bell MP Checkel JL Ackerman SJ McKean DJ. Biochemical and functional similarities between human eosinophil-derived neurotoxin and eosinophil cationic protein: homology with ribonuclease. Proc Natl Acad Sci USA (1986) 83:3146–50. doi: 10.1073/pnas.83.10.3146 PMC323469 3458170

[69] Rosenberg HF Domachowske JB. Eosinophils, ribonucleases and host defense: solving the puzzle. Immunol Res (1999) 20:261–74. doi: 10.1007/bf02790409 10741866

[70] Slifman NR Loegering Da McKean DJ Gleich GJ. Ribonuclease activity associated with human eosinophil-derived neurotoxin and eosinophil cationic protein. J Immunol (1986) 137:2913–7. doi: 10.4049/jimmunol.137.9.2913 3760576

[71] Abdul-Cader MS Amarasinghe A Abdul-Careem MF. Activation of toll-like receptor signaling pathways leading to nitric oxide-mediated antiviral responses. Arch Virol (2016) 161:2075–86. doi: 10.1007/s00705-016-2904-x PMC7087267 27233799

[72] Yousefi S Gold JA Andina N Lee JJ Kelly AM Kozlowski E. Catapult-like release of mitochondrial DNA by eosinophils contributes to antibacterial defense. Nat Med (2008) 14:949–53. doi: 10.1038/nm.1855 18690244

[73] Schönrich G Raftery MJ. Neutrophil extracellular traps go viral. Front Immunol (2016) 7:366. doi: 10.3389/fimmu.2016.00366 27698656 PMC5027205

[74] Wang H-B Ghiran I Matthaei K Weller PF. Airway eosinophils: allergic inflammation recruited professional antigen-presenting cells. J Immunol (2007) 179:7585–92. doi: 10.4049/jimmunol.179.11.7585 PMC2735454 18025204

[75] Woerly G Roger N Loiseau S Dombrowicz D Capron A Capron M. Expression of CD28 and CD86 by human eosinophils and role in the secretion of type 1 cytokines (interleukin 2 and interferon γ): inhibition by immunoglobulin a complexes. J Exp Med (1999) 190:487–95. doi: 10.1084/jem.190.4.487 PMC2195599 10449520

[76] Ohkawara Y Lim KG Xing Z Glibetic M Nakano K Dolovich J. CD40 expression by human peripheral blood eosinophils. J Clin Invest (1996) 97:1761–6. doi: 10.1172/JCI118603 PMC507241 8601642

[77] Samarasinghe AE Melo RCN Duan S LeMessurier KS Liedmann S Surman SL. Eosinophils promote antiviral immunity in mice infected with influenza a virus. J Immunol (2017) 198:3214–26. doi: 10.4049/jimmunol.1600787 PMC5384374 28283567

[78] Flores-Torres AS Salinas-Carmona MC Salinas E Rosas-Taraco AG. Eosinophils and respiratory viruses. Viral Immunol (2019) 32:198–207. doi: 10.1089/VIM.2018.0150 31140942

[79] Throsby M. CD11c + eosinophils in the murine thymus: developmental regulation and recruitment upon MHC class I-restricted thymocyte deletion. J Immunol (2020) 165(4):1965–75. doi: 10.4049/jimmunol.165.4.1965 10925279

[80] Syeda MZ Hong T Zhang C Ying S Shen H. Eosinophils: a friend or foe in human health and diseases. Kidney Dis (Basel) (2023) 9(1):26–38. doi: 10.1159/000528156 36756082 PMC9900469

[81] Borchers AT Chang C Gershwin ME Gershwin LJ. Respiratory syncytial virus - a comprehensive review. Clin Rev Allergy Immunol (2013) 45:331–79. doi: 10.1007/s12016-013-8368-9 PMC7090643 23575961

[82] Falsey AR. Respiratory syncytial virus infection in elderly and high-risk adults. N Engl J Med (2005) 352:1749–59. doi: 10.1056/nejmoa043951 15858184

[83] Domachowske JB Rosenberg HF. Respiratory syncytial virus infection: immune response, immunopathogenesis, and treatment. Clin Microbiol Rev (1999) 12:298–309. doi: 10.1128/cmr.12.2.298 10194461 PMC88919

[84] Del Pozo V. Eosinophils transcribe and translate messenger RNA for inducible nitric oxide. Immunol Lett (1997) 56:859–64. doi: 10.1016/s0165-2478(97)87469-x 8993004

[85] Levitz R Gao Y Dozmorov I Song R Wakeland EK Kahn JS. Distinct patterns of innate immune activation by clinical isolates of respiratory syncytial virus. PloS One (2017) 12:e0184318. doi: 10.1371/journal.pone.0184318 28877226 PMC5587315

[86] Percopo CM Dyer KD Ochkur SI Luo JL Fischer ER Lee JJ. Activated mouse eosinophils protect against lethal respiratory virus infection. Blood (2014) 123:743–52. doi: 10.1182/blood-2013-05-502443 PMC3907759 24297871

[87] Pennings JLA Schuurhof A Hodemaekers HM Buisman A de Rond LCGH Widjojoatmodjo MN. Systemic signature of the lung response to respiratory syncytial virus infection. PloS One (2011) 6:e21461. doi: 10.1371/journal.pone.0021461 21731757 PMC3123345

[88] Su Y-C Townsend D Herrero LJ Zaid A Rolph MS Gahan ME. Dual proinflammatory and antiviral properties of pulmonary eosinophils in respiratory syncytial virus vaccine-enhanced disease. J Virol (2015) 89:1564–78. doi: 10.1128/jvi.01536-14 PMC4300751 25410867

[89] Knudson CJ Hartwig SM Meyerholz DK Varga SM. RSV Vaccine-enhanced disease is orchestrated by the combined actions of distinct CD4 T cell subsets. PloS Pathog (2015) 11:e1004757. doi: 10.1371/journal.ppat.1004757 25769044 PMC4358888

[90] Prince GA Curtis SJ Yim KC Porter DD. Vaccine-enhanced respiratory syncytial virus disease in cotton rats following immunization with lot 100 or a newly prepared reference vaccine. J Gen Virol (2001) 82:2881–8. doi: 10.1099/0022-1317-82-12-2881 11714962

[91] Taubenberger JK Kash JC. Influenza virus evolution, host adaptation, and pandemic formation. Cell Host Microbe (2010) 7:440–51. doi: 10.1016/j.chom.2010.05.009 PMC2892379 20542248

[92] Bramley AM Dasgupta S Skarbinski J Kamimoto L Fry AM Finelli L. Intensive care unit patients with 2009 pandemic influenza a (H1N1pdm09) virus infection - united states, 2009. Influenza Other Respi Viruses (2012) 6:e134–e142. doi: 10.1111/j.1750-2659.2012.00385.x PMC4941711 22672249

[93] Eriksson CO Graham DA Uyeki TM Randolph AG. Risk factors for mechanical ventilation in U.S. children hospitalized with seasonal influenza and 2009 pandemic influenza a. Pediatr Crit Care Med (2012) 13:625–31. doi: 10.1097/PCC.0b013e318260114e PMC6615726 22895006

[94] McKenna JJ Bramley AM Skarbinski J Fry AM Finelli L Jain S. Asthma in patients hospitalized with pandemic influenza A(H1N1)pdm09 virus infection-united states, 2009. BMC Infect Dis (2013) 13:57. doi: 10.1186/1471-2334-13-57 23369034 PMC3585510

[95] Myles P Nguyen-Van-Tam JS Semple MG Brett SJ Bannister B Read RC. Differences between asthmatics and nonasthmatics hospitalised with influenza a infection. Eur Respir J (2013) 41:824–31. doi: 10.1183/09031936.00015512 PMC3612580 22903963

[96] Samarasinghe AE Woolard SN Boyd KL Hoselton SA Schuh JM Mccullers JA. The immune profile associated with acute allergic asthma accelerates clearance of influenza virus. Immunol Cell Biol (2014) 92:449–59. doi: 10.1038/icb.2013.113 PMC4037497 24469764

[97] Ishikawa H Sasaki H Fukui T. Mice with asthma are more resistant to influenza virus infection and NK cells activated by the induction of asthma have potentially protective effects. J Clin Immunol (2012) 32:256–67. doi: 10.1007/s10875-011-9619-2 PMC3305878 22134539

[98] Henrickson KJ. Parainfluenza viruses. Clin Microbiol Rev (2003) 16:242–64. doi: 10.1128/CMR.16.2.242-264.2003 PMC153148 12692097

[99] Johnston SL Pattemore PK Sanderson G Smith S Lampe F Josephs L. Community study of role of viral infections in exacerbations of asthma in 9-11 year old children. BMJ (1995) 310:1225–9. doi: 10.1136/bmj.310.6989.1225 PMC2549614 7767192

[100] Adamko DJ Yost BL Gleich GJ Fryer AD Jacoby DB. Ovalbumin sensitization changes the inflammatory response to subsequent parainfluenza infection: eosinophils mediate airway hyperresponsiveness, M2 muscarinic receptor dysfunction, and antiviral effects. J Exp Med (1999) 190:1465–77. doi: 10.1084/jem.190.10.1465 PMC2195693 10562321

[101] Lee JJ McGarry MP Farmer SC Denzler KL Larson KA Carrigan PE. Interleukin-5 expression in the lung epithelium of transgenic mice leads to pulmonary changes pathognomonic of asthma. J Exp Med (1997) 185:2143–56. doi: 10.1084/jem.185.12.2143 PMC2196351 9182686

[102] Drake MG Bivins-Smith ER Proskocil BJ Nie Z Scott GD Lee JJ. Human and mouse eosinophils have antiviral activity against parainfluenza virus. Am J Respir Cell Mol Biol (2016) 55:387–94. doi: 10.1165/rcmb.2015-0405OC PMC5023029 27049514

[103] Jacobs SE Lamson DM Kirsten S Walsh TJ. Human rhinoviruses. Clin Microbiol Rev (2013) 26:135–62. doi: 10.1128/CMR.00077-12 PMC3553670 23297263

[104] Handzel ZT Busse WW Sedgwick JB Vrtis R Lee WM Kelly EAB. Eosinophils bind rhinovirus and activate virus-specific T cells. J Immunol (1998) 160:1279–84. doi: 10.4049/jimmunol.160.3.1279 9570544

[105] Sabogal Piñeros YS Bal SM Dijkhuis A Majoor CJ Dierdorp BS Dekker T. Eosinophils capture viruses, a capacity that is defective in asthma. Allergy Eur J Allergy Clin Immunol (2019) 74:1898–909. doi: 10.1111/all.13802 PMC6852198 30934128

[106] Sabogal Piñeros YS Bal SM van de Pol MA Dierdorp BS Dekker T Dijkhuis A. Anti–IL-5 in mild asthma alters rhinovirus-induced macrophage, b-cell, and neutrophil responses (MATERIAL) a placebo-controlled, double-blind study. Am J Respir Crit Care Med (2019) 199:508–17. doi: 10.1164/RCCM.201803-0461OC 30192638

[107] Rosenberg HF Dyer KD Domachowske JB. Respiratory viruses and eosinophils: exploring the connections. Antiviral Res (2009) 83:1–9. doi: 10.1016/j.antiviral.2009.04.005 PMC2741084 19375458

[108] Yan B Yang J Xie Y Tang X. Relationship between blood eosinophil levels and COVID-19 mortality. World Allergy Organ J (2021) 14:100521. doi: 10.1016/j.waojou.2021.100521 33589865 PMC7877210

[109] Ryan FJ Hope CM Masavuli MG Lynn MA Mekonnen ZA Yeow AEL. Long-term perturbation of the peripheral immune system months after SARS-CoV-2 infection. BMC Med (2022) 20:26. doi: 10.1186/s12916-021-02228-6 35027067 PMC8758383

[110] Xie G Ding F Han L Yin D Lu H Zhang M. The role of peripheral blood eosinophil counts in COVID-19 patients. Allergy Eur J Allergy Clin Immunol (2021) 76:471–82. doi: 10.1111/all.14465 PMC7323233 32562554

[111] Roca E Ventura L Zattra CM Lombardi C Roca E. EOSINOPENIA: an early, effective and relevant COVID-19 biomarker? Qjm (2021) 114:68–9. doi: 10.1093/qjmed/hcaa259 PMC7499754 32877511

[112] Rosenberg HF Foster PS. Eosinophils and COVID-19: diagnosis, prognosis, and vaccination strategies. Semin Immunopathol (2021) 43:383–92. doi: 10.1007/s00281-021-00850-3 PMC7962927 33728484

[113] Karakonstantis S Gryllou N Papazoglou G Lydakis C. Eosinophil count (EC) as a diagnostic and prognostic marker for infection in the internal medicine department setting. Rom J Intern Med (2019) 57:166–74. doi: 10.2478/rjim-2018-0039 30517081

[114] Nair AP Soliman A Al Masalamani MA De Sanctis V Nashwan AJ Sasi S. Clinical outcome of eosinophilia in patients with covid-19: a controlled study. Acta BioMed (2020) 91:e2020165. doi: 10.23750/abm.v91i4.10564 33525219 PMC7927494

[115] Cauchois R Pietri L Dalmas JB Koubi M Capron T Cassir N. Eosinopenia as predictor of poor outcome in hospitalized COVID-19 adult patients from waves 1 and 2 of 2020 pandemic. Microorganisms (2022) 10:2423. doi: 10.3390/microorganisms10122423 36557676 PMC9783665

[116] Baicus C. White blood cells, COVID-19, and mendelian randomization. J Pers Med (2022) 12:18–21. doi: 10.3390/jpm12091425 PMC9500626 36143211

[117] Man MA Rajnoveanu RM Motoc NS Bondor CI Chis AF Lesan A. Neutrophil-to-lymphocyte ratio, platelets-to-lymphocyte ratio, and eosinophils correlation with high-resolution computer tomography severity score in COVID-19 patients. PloS One (2021) 16:e0252599. doi: 10.1371/journal.pone.0252599 34181675 PMC8238190

[118] González MM Gonzalo ES Lopez IC Fernández FA Pérez JLB Monge DM. The prognostic value of eosinophil recovery in COVID-19: a multicentre, retrospective cohort study on patients hospitalised in spanish hospitals. J Clin Med (2021) 10:305. doi: 10.3390/jcm10020305 33467585 PMC7830154

[119] Xiao A Zhao H Xia J Zhang L Zhang C Ruan Z. Triage modeling for differential diagnosis between COVID-19 and human influenza a pneumonia: classification and regression tree analysis. Front Med (2021) 8:673253. doi: 10.3389/fmed.2021.673253 PMC8382719 34447759

[120] Ma J Shi X Xu W Lv F Wu J Pan Q. Development and validation of a risk stratification model for screening suspected cases of COVID-19 in China. Aging (Albany NY) (2020) 12:13882–94. doi: 10.18632/aging.103694 PMC7425460 32727933

[121] Tordjman M Mekki A Mali RD Saab I Chassagnon G Guillo E. Pre-test probability for SARS-Cov-2-related infection score: the PARIS score. PloS One (2020) 15:e0243342. doi: 10.1371/journal.pone.0243342 33332360 PMC7745977

[122] Shen C Tan M Song X Zhang G Liang J Yu H. Comparative analysis of early-stage clinical features between COVID-19 and influenza a H1N1 virus pneumonia. Front Public Heal (2020) 8:206. doi: 10.3389/fpubh.2020.00206 PMC7243732 32574297

[123] Moghadas SM Fitzpatrick MC Shoukat A Zhang K Galvani AP. Simulated Identification of Silent COVID-19 Infections Among Children and Estimated Future Infection Rates With Vaccination. JAMA Netw Open (2021) 4(4):e217097. doi: 10.1001/jamanetworkopen.2021.7097 33890990 PMC8065378

[124] Gao YD Agache I Akdis M Nadeau K Klimek L Jutel M. The effect of allergy and asthma as a comorbidity on the susceptibility and outcomes of COVID-19. Int Immunol (2022) 34:177–88. doi: 10.1093/intimm/dxab107 PMC8689956 34788827

[125] Rodriguez L Pekkarinen PT Lakshmikanth T Tan Z Consiglio CR Pou C. Systems-level immunomonitoring from acute to recovery phase of severe COVID-19. Cell Rep Med (2020) 1:100078. doi: 10.1016/j.xcrm.2020.100078 32838342 PMC7405891

[126] Proal AD Vanelzakker MB Dobrovolny HM. Long COVID or post-acute sequelae of COVID-19 (PASC): an overview of biological factors that may contribute to persistent symptoms. Front Microbiol (2021) 12:698169. doi: 10.3389/fmicb.2021.698169 34248921 PMC8260991

[127] Phetsouphanh C Darley DR Wilson DB Howe A Munier CML Patel SK. Immunological dysfunction persists for 8 months following initial mild-to-moderate SARS-CoV-2 infection. (2022) 23(2):210–6. doi: 10.1038/s41590-021-01113-x 35027728

[128] Arish M Qian W. COVID - 19 immunopathology: from acute diseases to chronic sequelae. J Med Virol (2023) 95(1):e28122. doi: 10.1002/jmv.28122 36056655 PMC9537925

[129] Davis HE Mccorkell L Vogel JM Topol EJ. Long COVID: major findings, mechanisms and recommendations. Nat Rev Microbiol (2023) 21:133–46. doi: 10.1038/s41579-022-00846-2 PMC9839201 36639608

[130] Leon SL Ostrosky TW Perelman C Sepulveda R Rebolledo PA Cuapio A. More than 50 long − term effects of COVID − 19: a systematic review and meta − analysis middle East respiratory syndrome. Sci Rep (2021) 11(1):16144. doi: 10.1038/s41598-021-95565-8 34373540 PMC8352980

[131] Gebremeskel S Schanin J Coyle KM Butuci M Luu T Brock EC. Mast cell and eosinophil activation are associated with COVID-19 and TLR-mediated viral inflammation: implications for an anti-Siglec-8 antibody. Front Immunol (2021) 12:650331. doi: 10.3389/fimmu.2021.650331 33777047 PMC7988091

[132] Jukema BN Smit K Hopman MTE Bongers CCWG Pelgrim TC. Neutrophil and eosinophil responses remain abnormal for several months in primary care patients with COVID-19 disease. Nature Reviews Microbiology (2022) (21):133–46. doi: 10.3389/falgy.2022.942699 PMC9365032 35966226

[133] Costa V Gal A De Menezes DC De Lima L Regina V Pal M. Evaluation of the hematological patterns from up to 985 days. Viruses (2023) 15(4):879. doi: 10.3390/v15040879 37112859 PMC10142608

[134] Laorden D Domínguez-ortega J Carpio C Barranco P Romero D Quirce S. Long COVID outcomes in an asthmatic cohort and its implications for asthma control. Front Allergy (2023) 3:942699. doi: 10.1016/j.rmed.2022.107092 PMC9755318 36535372

[135] Klein J Wood J Jaycox J Lu P Dhodapkar RM Gehlhausen JR. Distinguishing features of Long COVID identified through immune profiling. medRxiv : the preprint server for health sciences, 2022.08.09.22278592. doi: 10.1101/2022.08.09.22278592 PMC10620090 37748514

[136] Lindsley AW Schwartz JT Rothenberg ME. Eosinophil responses during COVID-19 infections and coronavirus vaccination. J Allergy Clin Immunol (2020) 146:1–7. doi: 10.1016/j.jaci.2020.04.021 32344056 PMC7194727

[137] Nakagome K Nagata M. Innate immune responses by respiratory viruses, including rhinovirus, during asthma exacerbation. Front Immunol (2022) 13:865973. doi: 10.3389/fimmu.2022.865973 35795686 PMC9250977

[138] Andreone S Spadaro F Buccione C Mancini J Tinari A Sestili P. IL-33 promotes CD11b/CD18-mediated adhesion of eosinophils to cancer cells and synapse-polarized degranulation leading to tumor cell killing. Cancers (Basel) (2019) 11(11):1664. doi: 10.3390/cancers11111664 31717819 PMC6895824

[139] Zeng S Wu J Liu J Qi F Liu B. IL-33 receptor (ST2) signalling is important for regulation of Th2-mediated airway inflammation in a murine model of acute respiratory syncytial virus infection. Scand J Immunol (2015) 81:494–502. doi: 10.1111/sji.12284 25721734

[140] Liu J Wu J Qi F Zeng S Xu L Hu H. Natural helper cells contribute to pulmonary eosinophilia by producing IL-13 *via* IL-33/ST2 pathway in a murine model of respiratory syncytial virus infection. Int Immunopharmacol (2015) 28:337–43. doi: 10.1016/j.intimp.2015.05.035 26044350

[141] Schaunaman N Sanchez A Dimasuay KG Pavelka N Numata M Alam R. Interleukin 1 receptor-like 1 (IL1RL1) promotes airway bacterial and viral infection and inflammation. Infect Immun (2019) 87:e00340–19. doi: 10.1128/IAI.00340-19 PMC6589056 31061143

[142] Dill-McFarland KA Schwartz JT Zhao H Shao B Fulkerson PC Altman MC. Eosinophil-mediated suppression and anti–IL-5 enhancement of plasmacytoid dendritic cell interferon responses in asthma. J Allergy Clin Immunol (2022) 150:666–75. doi: 10.1016/j.jaci.2022.03.025 35413355

[143] Mathur SK Fichtinger PS Kelly JT Lee WM Gern JE Jarjour NN. Interaction between allergy and innate immunity: model for eosinophil regulation of epithelial cell interferon expression. Ann Allergy Asthma Immunol (2013) 111:25–31.el. doi: 10.1016/j.anai.2013.05.010 PMC3708694 23806456

[144] Porsbjerg C Nieto-Fontarigo JJ Cerps S Ramu S Menzel M Hvidtfeldt M. Phenotype and severity of asthma determines bronchial epithelial immune responses to a viral mimic. Eur Respir J (2022) 60:2102333. doi: 10.1183/13993003.02333-2021 34916261

[145] Jartti T Gern JE. Role of viral infections in the development and exacerbation of asthma in children. J Allergy Clin Immunol (2017) 140:895–906. doi: 10.1016/j.jaci.2017.08.003 28987219 PMC7172811

[146] Zhu J Message SD Mallia P Kebadze T Contoli M Ward CK. Bronchial mucosal IFN-α/β and pattern recognition receptor expression in patients with experimental rhinovirus-induced asthma exacerbations. J Allergy Clin Immunol (2019) 143:114. doi: 10.1016/J.JACI.2018.04.003 29698627 PMC6320262

[147] Mehta AK Croft M. Rhinovirus infection promotes eosinophilic airway inflammation after prior exposure to house dust mite allergen. ImmunoHorizons (2020) 4:498–507. doi: 10.4049/immunohorizons.2000052 32792363

[148] Warren KJ Poole JA Sweeter JM DeVasure JM Dickinson JD Peebles RS. Neutralization of IL-33 modifies the type 2 and type 3 inflammatory signature of viral induced asthma exacerbation. Respir Res (2021) 22:206. doi: 10.1186/s12931-021-01799-5 34266437 PMC8281667

[149] Altman MC Gill MA Whalen E Babineau DC Shao B Liu AH. Transcriptome networks identify mechanisms of viral and nonviral asthma exacerbations in children. Nat Immunol (2019) 20:637–51. doi: 10.1038/S41590-019-0347-8 PMC6472965 30962590

[150] Mihaylova VT Kong Y Fedorova O Sharma L Dela Cruz CS Pyle AM. Regional differences in airway epithelial cells reveal tradeoff between defense against oxidative stress and defense against rhinovirus. Cell Rep (2018) 24:3000–3007.e3. doi: 10.1016/J.CELREP.2018.08.033 30208323 PMC6190718

[151] Wark PAB Johnston SL Bucchieri F Powell R Puddicombe S Laza-Stanca V. Asthmatic bronchial epithelial cells have a deficient innate immune response to infection with rhinovirus. J Exp Med (2005) 201:937–47. doi: 10.1084/jem.20041901 PMC2213100 15781584

[152] Contoli M Message SD Laza-Stanca V Edwards MR Wark PAB Bartlett NW. Role of deficient type III interferon-λ production in asthma exacerbations. Nat Med (2006) 12:1023–6. doi: 10.1038/nm1462 16906156

[153] Durrani SR Montville DJ Pratt AS Sahu S Devries MK Rajamanickam V. Innate immune responses to rhinovirus are reduced by the high-affinity IgE receptor in allergic asthmatic children. J Allergy Clin Immunol (2012) 130:489–95. doi: 10.1016/j.jaci.2012.05.023 PMC3437329 22766097

[154] Ramakrishnan RK Al Heialy S Hamid Q. Implications of preexisting asthma on COVID-19 pathogenesis. Am J Physiol - Lung Cell Mol Physiol (2021) 320:L880–1. doi: 10.1152/ajplung.00547.2020 PMC8143784 33759572

[155] Adir Y Saliba W Beurnier A Humbert M. Asthma and COVID-19: an update. Eur Respir Rev (2021) 30:210152. doi: 10.1183/16000617.0152-2021 34911694 PMC8674937

[156] Eggert LE He Z Collins W Lee AS Dhondalay G Jiang SY. Asthma phenotypes, associated comorbidities, and long-term symptoms in COVID-19. Allergy (2022) 77:173. doi: 10.1111/ALL.14972 34080210 PMC8222896

[157] Ferastraoaru D Hudes G Jerschow E Jariwala S Karagic M de Vos G. Eosinophilia in asthma patients is protective against severe COVID-19 illness. J Allergy Clin Immunol Pract (2021) 9:1152. doi: 10.1016/J.JAIP.2020.12.045 33495097 PMC7826039

[158] Drake MG Fryer AD Jacoby DB. Protective effects of eosinophils against COVID-19: more than an ACE(2) in the hole? J Allergy Clin Immunol Pract (2021) 9:2539–40. doi: 10.1016/j.jaip.2021.02.062 PMC8181741 34112482

[159] Lombardi C Bagnasco D Passalacqua G. COVID-19, eosinophils, and biologicals for severe asthma. Front Allergy (2022) 3:859376. doi: 10.3389/falgy.2022.859376 35769563 PMC9234863

[160] Jesenak M Banovcin P Diamant Z. COVID-19, chronic inflammatory respiratory diseases and eosinophils–observations from reported clinical case series. Allergy Eur J Allergy Clin Immunol (2020) 75:1819–22. doi: 10.1111/all.14353 32369190

[161] Vitte J Diallo AB Boumaza A Lopez A Michel M Allardet-Servent J. A granulocytic signature identifies COVID-19 and its severity. J Infect Dis (2020) 222:1985–96. doi: 10.1093/infdis/jiaa591 PMC7543529 32941618

[162] Gao Y Cai L Li L Zhang Y Li J Luo C. Emerging effects of IL-33 on COVID-19. Int J Mol Sci (2022) 23:13656. doi: 10.3390/ijms232113656 36362440 PMC9658128

[163] Zizzo G Cohen PL. Imperfect storm: is interleukin-33 the Achilles heel of COVID-19? Lancet Rheumatol (2020) 2:e779–90. doi: 10.1016/S2665-9913(20)30340-4 PMC7546716 33073244

[164] Cayrol C Girard JP. Interleukin-33 (IL-33): a critical review of its biology and the mechanisms involved in its release as a potent extracellular cytokine. Cytokine (2022) 156:155891. doi: 10.1016/j.cyto.2022.155891 35640416

[165] Stanczak MA Sanin DE Apostolova P Nerz G Lampaki D Hofmann M. IL-33 expression in response to SARS-CoV-2 correlates with seropositivity in COVID-19 convalescent individuals. Nat Commun (2021) 12:2133. doi: 10.1038/s41467-021-22449-w 33837219 PMC8035172

[166] Rodig SJ Kutok JL. Bone marrow disorders with associated eosinophilia. Diagn Histopathol (2009) 15:107–15. doi: 10.1016/J.MPDHP.2009.01.012

[167] Yamasaki A Okazaki R Harada T. Neutrophils and asthma. Diagnostics (2022) 12:1175. doi: 10.3390/diagnostics12051175 35626330 PMC9140072

[168] Sutton JM. Urinary eosinophils. Arch Intern Med (1986) 146:2243–4. doi: 10.1001/ARCHINTE.1986.00360230183026 3535716

[169] Wiszniewska M Pas-Wyroslak A Palczynski C Walusiak-Skorupa J. Eosinophilia in conjunctival tear fluid among patients with pollen allergy. Ann Allergy Asthma Immunol (2011) 107:281–2. doi: 10.1016/j.anai.2011.06.002 21875549

[170] Dixon DL Forsyth KD. Leukocytes in expressed breast milk of asthmatic mothers. Allergol Immunopathol (Madr) (2017) 45:325–32. doi: 10.1016/j.aller.2016.08.015 27889334

[171] Kaya D Demirezen S Beksaç M. The presence of eosinophil leucocytes in cervicovaginal smears with actinomyces-like organisms: light microscopic examination. J Cytol (2012) 29:226. doi: 10.4103/0970-9371.103939 23326024 PMC3543589

[172] Bosch I Oehmichen M. Eosinophilic granulocytes in cerebrospinal fluid: analysis of 94 cerebrospinal fluid specimens and review of the literature. J Neurol (1978) 219:93–105. doi: 10.1007/BF00314392/METRICS 81295

[173] Kay AB. The early history of the eosinophil. Clin Exp Allergy (2015) 45:575–82. doi: 10.1111/cea.12480 25544991

[174] Gulati G Song J Florea AD Gong J. Purpose and criteria for blood smear scan, blood smear examination, and blood smear review. Ann Lab Med (2013) 33:1–7. doi: 10.3343/ALM.2013.33.1.1 PMC3535191 23301216

[175] Bain BJ Bates I Laffan MA. Dacie and Lewis practical haematology. e-Book:&nbsp;Expert consult: online and print. (2016).

[176] Blumenreich MS. The white blood cell and differential count. In: Walker HK Hall WD Hurst JW editors. Clinical Methods: The History, Physical, and Laboratory Examinations. 3rd ed. Boston: ButterworthsChapter 153. (1990). 21250045

[177] Bain B Bates I Laffan MA. Dacie and Lewis Practical Haematology, 12th Edition - August 11, 2016. Elsevier.

[178] Buttarello M Plebani M. Automated blood cell counts: state of the art. Am J Clin Pathol (2008) 130(1):104–16. doi: 10.1309/EK3C7CTDKNVPXVTN 18550479

[179] Cherian S Levin G Lo WY Mauck M Kuhn D Lee C. Evaluation of an 8-color flow cytometric reference method for white blood cell differential enumeration. Cytom Part B - Clin Cytom (2010) 78:319–28. doi: 10.1002/cyto.b.20529 20533390

[180] Roussel M Davis BH Fest T Wood BL. Toward a reference method for leukocyte differential counts in blood: comparison of three flow cytometric candidate methods. Cytom Part A (2012) 81A:973–82. doi: 10.1002/CYTO.A.22092 22736499

[181] Hansel TT De Vries IJM Iff T Rihs S Wandzilak M Betz S. An improved immunomagnetic procedure for the isolation of highly purified human blood eosinophils. J Immunol Methods (1991) 145:105–10. doi: 10.1016/0022-1759(91)90315-7 1662676

[182] Lim KG Weller PF. Isolation of human eosinophils. Curr Protoc Immunol (1996) 20. doi: 10.1002/0471142735.im0731s20 PMC3420812 18432843

[183] Munoz NM Leff AR. Highly purified selective isolation of eosinophils from human peripheral blood by negative immunomagnetic selection. Nat Protoc 2006 16 (2007) 1:2613–20. doi: 10.1038/nprot.2006.340 17406516

[184] Wacht G Poirot A Charles AL Radosavljevic M Uring-Lambert B de Blay F. FACS - based isolation of human eosinophils allows purification of high quality RNA. J Immunol Methods (2018) 463:47–53. doi: 10.1016/J.JIM.2018.09.003 30217720

[185] Review P The J-Su F. (1995) 51:3728–38. of a at.

[186] Thurau AM Schulz U Wolf V Krug N Schauer U. Identification of eosinophils by flow cytometry. Cytometry (1996) 23:150–8. doi: 10.1002/(SICI)1097-0320(19960201)23:2<150::AID-CYTO8>3.0.CO;2-O 8742174

[187] Ross R Klebanoff SJ. The eosinophilic leukocyte. fine structure studies of changes in the uterus during the estrous cycle. J Exp Med (1966) 124:653–60. doi: 10.1084/JEM.124.4.653 PMC2138249 5950887

[188] Wu D Molofsky AB Liang HE Ricardo-Gonzalez RR Jouihan HA Bando JK. Eosinophils sustain adipose alternatively activated macrophages associated with glucose homeostasis. Science (80-) (2011) 332:243–7. doi: 10.1126/SCIENCE.1201475 PMC3144160 21436399

[189] Xenakis JJ Howard ED Smith KM Olbrich CL Huang Y Anketell D. Resident intestinal eosinophils constitutively express antigen presentation markers and include two phenotypically distinct subsets of eosinophils. Immunology (2018) 154:298–308. doi: 10.1111/imm.12885 29281125 PMC5980140

[190] Curto E Mateus-Medina ÉF Crespo-Lessmann A Osuna-Gómez R Ujaldón-Miró C García-Moral A. Identification of two eosinophil subsets in induced sputum from patients with allergic asthma according to CD15 and CD66b expression. Int J Environ Res Public Health (2022) 19:1–10. doi: 10.3390/ijerph192013400 PMC9602830 36293979

[191] Kanda A Yun Y Van Bui D Nguyen LM Kobayashi Y Suzuki K. Corrigendum to “The multiple functions and subpopulations of eosinophils in tissues under steady-state and pathological conditions” [Allergol int 70 (2021) 9–18] (Allergology international (2021) 70(1) (9–18), (S1323893020301441), (10.1016/j.alit.2020.11. Allergol Int (2021) 70:277. doi: 10.1016/j.alit.2021.01.002 33589365

[192] Valent P Degenfeld-Schonburg L Sadovnik I Horny HP Arock M Simon HU. Eosinophils and eosinophil-associated disorders: immunological, clinical, and molecular complexity. Semin Immunopathol (2021) 43:423–38. doi: 10.1007/s00281-021-00863-y PMC8164832 34052871

[193] Zimmermann N Conkright JJ Rothenberg ME. CC chemokine receptor-3 undergoes prolonged ligand-induced internalization. J Biol Chem (1999) 274:12611–8. doi: 10.1074/JBC.274.18.12611 10212240

[194] Kaplan AP. Chemokines, chemokine receptors and allergy. Int Arch Allergy Immunol (2001) 124(4):423–31. doi: 10.1159/000053777 11340325

[195] Uguccioni M Mackay CR Ochensberger B Loetscher P Rhis S LaRosa GJ. High expression of the chemokine receptor CCR3 in human blood basophils. role in activation by eotaxin, MCP-4, and other chemokines. J Clin Invest (1997) 100:1137–43. doi: 10.1172/JCI119624 PMC508288 9276730

[196] Gustafson MP Lin Y Maas ML Van Keulen VP Johnston PB Peikert T. A method for identification and analysis of non-overlapping myeloid immunophenotypes in humans. PloS One (2015) 10:1–19. doi: 10.1371/journal.pone.0121546 PMC4370675 25799053

[197] Hirai H Tanaka K Yoshie O Ogawa K Kenmotsu K Takamori Y. Prostaglandin D2 selectively induces chemotaxis in T helper type 2 cells, eosinophils, and basophils via seven-transmembrane receptor CRTH2. J Exp Med (2001) 193:255–61. doi: 10.1084/jem.193.2.255 PMC2193345 11208866

[198] Youngblood BA Leung J Falahati R Williams J Schanin J Brock EC. Discovery, function, and therapeutic targeting of siglec-8. Cells (2021) 10:1–14. doi: 10.3390/cells10010019 PMC7823959 33374255

[199] Johansson MW Kelly EA Nguyen CL Jarjour NN Bochner BS. Characterization of siglec-8 expression on lavage cells after segmental lung allergen challenge. Int Arch Allergy Immunol (2018) 177:16. doi: 10.1159/000488951 29879704 PMC6105496

[200] Yu YRA Hotten DF Malakhau Y Volker E Ghio AJ Noble PW. Flow cytometric analysis of myeloid cells in human blood, bronchoalveolar lavage, and lung tissues. Am J Respir Cell Mol Biol (2016) 54:13–24. doi: 10.1165/rcmb.2015-0146OC 26267148 PMC4742930

[201] Solovjov DA Pluskota E Plow EF. Distinct roles for the alpha and beta subunits in the functions of integrin alphaMbeta2. J Biol Chem (2005) 280:1336–45. doi: 10.1074/JBC.M406968200 15485828

[202] Yoon J Terada A Kita H. CD66b regulates adhesion and activation of human eosinophils. J Immunol (2007) 179:8454–62. doi: 10.4049/jimmunol.179.12.8454 18056392

[203] Matucci A Nencini F Maggiore G Chiccoli F Accinno M Vivarelli E. High proportion of inflammatory CD62Llow eosinophils in blood and nasal polyps of severe asthma patients. Clin Exp Allergy (2023) 53(1):78–87. doi: 10.1111/CEA.14153 35490414

[204] Davenpeck KL Brummet ME Hudson SA Mayer RJ Bochner BS. Activation of human leukocytes reduces surface p-selectin glycoprotein ligand-1 (PSGL-1, CD162) and adhesion to p-selectin *In vitro* . J Immunol (2000) 165:2764–72. doi: 10.4049/jimmunol.165.5.2764 10946308

[205] Na HJ Hamilton RG Klion AD Bochner BS. Biomarkers of eosinophil involvement in allergic and eosinophilic diseases: review of phenotypic and serum markers including a novel assay to quantify levels of soluble siglec-8. J Immunol Methods (2012) 383:39–46. doi: 10.1016/j.jim.2012.05.017 22683541 PMC3411856

[206] Nopp A Lundahl J Halldén G. Quantitative, rather than qualitative, differences in CD69 upregulation in human blood eosinophils upon activation with selected stimuli. Allergy Eur J Allergy Clin Immunol (2000) 55:148–56. doi: 10.1034/j.1398-9995.2000.00363.x 10726729

[207] Mawhorter SD Stephany DA Ottesen EA Nutman TB. Identification of surface molecules associated with physiologic activation of eosinophils. application of whole-blood flow cytometry to eosinophils. J Immunol (1996) 156:4851–8. doi: 10.4049/jimmunol.156.12.4851 8648134

[208] Tran TAT Grievink HW Lipinska K Kluft C Burggraaf J Moerland M. Whole blood assay as a model for *in vitro* evaluation of inflammasome activation and subsequent caspase-mediated interleukin-1 beta release. PloS One (2019) 14:1–16. doi: 10.1371/journal.pone.0214999 PMC6453527 30958862

[209] Suzukawa M Koketsu R Iikura M Nakae S Matsumoto K Nagase H. Interleukin-33 enhances adhesion, CD11b expression and survival in human eosinophils. Lab Investig (2008) 88:1245–53. doi: 10.1038/labinvest.2008.82 18762778

[210] Davoine F Sim A Wierzbicki T Leong C Puttagunta L McGaw T. Human eosinophils express granzyme b and perforin: potential role in tumour killing in oral squamous cancer. J Allergy Clin Immunol (2006) 117:S15. doi: 10.1016/j.jaci.2005.12.064

[211] Lintomen L Franchi G Nowill A Condino-Neto A de Nucci G Zanesco A. Human eosinophil adhesion and degranulation stimulated with eotaxin and RANTES *in vitro*: lack of interaction with nitric oxide. BMC Pulm Med (2008) 8:13. doi: 10.1186/1471-2466-8-13 18700028 PMC2527293

[212] Lourda M Dzidic M Hertwig L Bergsten H Palma Medina LM Sinha I. High-dimensional profiling reveals phenotypic heterogeneity and disease-specific alterations of granulocytes in COVID-19. Proc Natl Acad Sci USA (2021) 118:1–12. doi: 10.1073/pnas.2109123118 PMC8501786 34548411

[213] Spijkerman R Hesselink L Hellebrekers P Vrisekoop N Hietbrink F Leenen LPH. Automated flow cytometry enables high performance point-of-care analysis of leukocyte phenotypes. J Immunol Methods (2019) 474:1–13. doi: 10.1016/J.JIM.2019.112646 31419409

[214] Maecker HT McCoy JP Amos M Elliott J Gaigalas A Wang L. A model for harmonizing flow cytometry in clinical trials. Nat Immunol (2010) 11:975–8. doi: 10.1038/ni1110-975 PMC3400260 20959798

[215] Macchia I La Sorsa V Ruspantini I Sanchez M Tirelli V Carollo M. Multicentre harmonisation of a six-colour flow cytometry panel for Naïve/Memory T cell immunomonitoring. J Immunol Res (2020) 2020:1–15. doi: 10.1155/2020/1938704 PMC7153001 32322591

[216] Streitz M Miloud T Kapinsky M Reed MR Magari R Geissler EK. Standardization of whole blood immune phenotype monitoring for clinical trials: panels and methods from the ONE study. Transplant Res (2013) 2(1):17. doi: 10.1186/2047-1440-2-17 24160259 PMC3827923

[217] Jamin C Le Lann L Alvarez-Errico D Barbarroja N Cantaert T Ducreux J. Multi-center harmonization of flow cytometers in the context of the European “PRECISESADS” project. Autoimmun Rev (2016) 15:1038–45. doi: 10.1016/j.autrev.2016.07.034 27490203

[218] Sriaroon P Ballow M. Biological modulators in eosinophilic diseases. Clin Rev Allergy Immunol (2014) 50:252–72. doi: 10.1007/S12016-014-8444-9 25129490

[219] Jiao Q Zhou A Shi C Fan Y Zheng Y Wang J. Involvement of CD40-CD40L and ICOS-ICOSL pathways in the development of chronic rhinosinusitis by modulating eosinophil function. Authorea Prepr (2022). doi: 10.22541/AU.167064127.72955313/V1 PMC10267736 37325657

[220] Cabrera López C Sánchez Santos A Lemes Castellano A Cazorla Rivero S Breña Atienza J González Dávila E. Eosinophils sub-types in asthmatic and COPD patients. Am J Respir Crit Care Med (2023) Online ahead of print. doi: 10.1164/rccm.202301-0149OC 37071848

[221] Vitiello L Mazzuca C Finamore P Zotti S Travaglini S Incalzi RA. Blood eosinophils activation type, besides count, associate with exacerbations in asthma. Eur Respir J (2022) 60:2752. doi: 10.1183/13993003.CONGRESS-2022.2752

[222] Salas LA Zhang Z Koestler DC Butler RA Hansen HM Molinaro AM. Enhanced cell deconvolution of peripheral blood using DNA methylation for high-resolution immune profiling. Nat Commun (2022) 13:1–13. doi: 10.1038/s41467-021-27864-7 PMC8828780 35140201

[223] Fettrelet T Gigon L Karaulov A Yousefi S Simon HU. The enigma of eosinophil degranulation. Int J Mol Sci (2021) 22:1–19. doi: 10.3390/ijms22137091 PMC8268949 34209362

[224] Bozeman P Learn D Thomas E. Assay of the human leukocyte enzymes myeloperoxidase and eosinophil peroxidase. J Immunol Methods (1990) 126(1):125–33. doi: 10.1016/0022-1759(90)90020-V 2154520

[225] Grosicki M Adami M Micheloni C Głuch-Lutwin M Siwek A Latacz G. Eosinophils adhesion assay as a tool for phenotypic drug screening - the pharmacology of 1,3,5 – triazine and 1H-indole like derivatives against the human histamine H4 receptor. Eur J Pharmacol (2021) 890:1–12. doi: 10.1016/j.ejphar.2020.173611 33017589

[226] Peters MC Wenzel SE. Intersection of biology and therapeutics: type 2 targeted therapeutics for adult asthma. Lancet (2020) 395:371–83. doi: 10.1016/S0140-6736(19)33005-3 PMC8522504 32007172

[227] Brusselle G Pavord ID Landis S Pascoe S Lettis S Morjaria N. Blood eosinophil levels as a biomarker in COPD. Respir Med (2018) 138:21–31. doi: 10.1016/j.rmed.2018.03.016 29724389

[228] Pavord ID. Blood eosinophil–directed management of airway disease the past, present, and future. Am J Respir Crit Care Med (2020) 202:637–9. doi: 10.1164/rccm.202004-1013ED PMC7462396 32356667

[229] Wechsler ME Munitz A Ackerman SJ Drake MG Jackson DJ Wardlaw AJ. Eosinophils in health and disease: a state-of-the-Art review. Mayo Clin Proc (2021) 96:2694–707. doi: 10.1016/j.mayocp.2021.04.025 34538424

[230] Tashkin DP Wechsler ME. Role of eosinophils in airway inflammation of chronic obstructive pulmonary disease. Int J COPD (2018) 13:335–49. doi: 10.2147/COPD.S152291 PMC5777380 29403271

